# The kisspeptin-1 receptor antagonist peptide-234 aggravates uremic cardiomyopathy in a rat model

**DOI:** 10.1038/s41598-023-41037-0

**Published:** 2023-08-28

**Authors:** Hoa Dinh, Zsuzsanna Z. A. Kovács, Fanni Márványkövi, Merse Kis, Klaudia Kupecz, Gergő Szűcs, Marah Freiwan, Gülsüm Yilmaz Lauber, Eylem Acar, Andrea Siska, Katalin Eszter Ibos, Éva Bodnár, András Kriston, Ferenc Kovács, Péter Horváth, Imre Földesi, Gábor Cserni, Bruno K. Podesser, Peter Pokreisz, Attila Kiss, László Dux, Krisztina Csabafi, Márta Sárközy

**Affiliations:** 1 Department of Biochemistry and Interdisciplinary Centre of Excellence, Albert Szent-Györgyi Medical School, University of Szeged, 6720 Szeged, Hungary; 2 Department of Biochemistry, Bach Mai Hospital, Hanoi, 100000 Vietnam; 3 Ludwig Boltzmann Institute for Cardiovascular Research at Center for Biomedical Research and Translational Surgery, Medical University of Vienna, A1090 Vienna, Austria; 4 Department of Laboratory Medicine, Albert Szent-Györgyi Medical School, University of Szeged, 6720 Szeged, Hungary; 5 Department of Pathophysiology, Albert Szent-Györgyi Medical School, University of Szeged, Szeged, 6720 Hungary; 6 Synthetic and Systems Biology Unit, Biological Research Centre, Eötvös Loránd Research Network, 6726 Szeged, Hungary; 7 Single-Cell Technologies Ltd, Szeged, 6726 Hungary; 8 Institute for Molecular Medicine Finland (FIMM), University of Helsinki, 00014 Helsinki, Finland; 9 Department of Pathology, Albert Szent-Györgyi Medical School, University of Szeged, Szeged, 6720 Hungary

**Keywords:** Experimental models of disease, Molecular medicine, Chronic kidney disease, Cardiac hypertrophy, Cardiovascular diseases

## Abstract

Uremic cardiomyopathy is characterized by diastolic dysfunction, left ventricular hypertrophy (LVH), and fibrosis. Dysregulation of the kisspeptin receptor (KISS1R)-mediated pathways are associated with the development of fibrosis in cancerous diseases. Here, we investigated the effects of the KISS1R antagonist peptide-234 (P234) on the development of uremic cardiomyopathy. Male Wistar rats (300–350 g) were randomized into four groups: (i) Sham, (ii) chronic kidney disease (CKD) induced by 5/6 nephrectomy, (iii) CKD treated with a lower dose of P234 (*ip.* 13 µg/day), (iv) CKD treated with a higher dose of P234 (*ip.* 26 µg/day). Treatments were administered daily from week 3 for 10 days. At week 13, the P234 administration did not influence the creatinine clearance and urinary protein excretion. However, the higher dose of P234 led to reduced anterior and posterior wall thicknesses, more severe interstitial fibrosis, and overexpression of genes associated with left ventricular remodeling (*Ctgf, Tgfb, Col3a1, Mmp9*), stretch (*Nppa*), and apoptosis (*Bax, Bcl2, Casp7*) compared to the CKD group. In contrast, no significant differences were found in the expressions of apoptosis-associated proteins between the groups. Our results suggest that the higher dose of P234 hastens the development and pathophysiology of uremic cardiomyopathy by activating the fibrotic TGF-β-mediated pathways.

## Introduction

Chronic kidney disease (CKD) is a major public health problem affecting over 10% of the population worldwide due to the growing prevalence of its primary causes, including diabetes mellitus, hypertension, and aging^1,2^. CKD and end-stage renal disease carry high morbidity and mortality rates, primarily driven by concomitant cardiovascular diseases (CVDs), including heart failure^3,4^. CKD-associated chronic and often irreversible structural and functional changes of the heart are called uremic cardiomyopathy^5,6^, characterized by diastolic dysfunction, left ventricular hypertrophy (LVH), and cardiac fibrosis in CKD patients^4,7,8^. LVH and cardiac fibrosis commonly involve the activation and proliferation of cardiac fibroblasts and the expansion of extracellular matrix, including collagen isoforms, leading to distorted cardiac structure with diastolic and systolic dysfunction^3,9^. This pathologic cardiac remodeling leads to compensatory LVH and, consequently, to heart failure with preserved ejection fraction (HFpEF)^4,10^. Later, the fibrotic process becomes more prominent, leading to tissue hypoxia and the activation of cell death mechanisms, including apoptosis in CKD^4,7^. These molecular mechanisms result in left ventricular wall thinning, chamber dilation, and, without intervention, ultimately cause the progression of pathologic cardiac remodeling to heart failure with reduced ejection fraction (HFrEF)^4,9^.

Multiple mechanisms may contribute to the development of uremic cardiomyopathy, including non-CKD specific factors, such as hypertension, hemodynamic overload, overactivation of the renin–angiotensin–aldosterone system (RAAS), and sympathetic nervous system, endothelial dysfunction, inflammation, and increased nitro-oxidative stress, as well as CKD-specific factors, including circulating uremic toxins and renal anemia^4,8^. However, the complex underlying mechanisms of uremic cardiomyopathy remain unclarified, and the current treatment options are insufficient to improve the poor outcome of CKD patients. Therefore, elucidating novel mechanisms in the development of uremic cardiomyopathy is crucial to discover new drug targets to decrease the burden of cardiovascular morbidity and mortality in CKD patients.

The KISS1 gene is a metastasis-suppressor gene discovered in melanoma cells^11^. It encodes a 145 amino acid precursor protein, which is cleaved into shorter kisspeptins (KPs) of 10, 13, 14, or 54 amino acids in length in the blood by matrix metalloproteinases (MMPs) such as MMP-9^11^. All KPs can activate the kisspeptin receptor (KISS1R), a G protein-coupled receptor (previously known as GPR54)^11,12^. After KPs bind to the KISS1R, the phosphorylated Gq/11 will activate phospholipase C (PLC), leading to calcium ion (Ca^2+^) mobilization^13,14^. Next to the Gq-PLC-Ca^2+^ pathway, however, other intracellular transduction pathways are also recruited, some of which are cell-type specific^15^. In point of fact, KISS1R-mediated ERK1/2 pathway activation was found in hypothalamic explants and CHO-K1 cells; however, in oxytocin neurons, KP failed to affect ERK1/2^16^. The activation of the ERK1/2 signaling plays an important role in the development of cardiac hypertrophy and protects against apoptosis^17^. However, no data on the KISS1R-mediated ERK1/2 activation in the cardiovascular system is available. Furthermore, p38 and phosphatidylinositol 3‑kinase (PI3K)/protein kinase B (AKT) pathways were found to mediate KP’s action in thyroid cancer cells and preoptic neurons but not in luteal cells^15^.

KPs regulate many biological processes, including tumor growth and metastasis, metabolism, puberty onset, and reproductive functions^13,14,18,19^. The KISS1/KISS1R signaling pathway was reported to have anti-metastasis/anti-tumorigenic roles in many cancer types^18^. The tumor-suppressive role of the KISS1R was mainly linked to the inhibition of tumor invasion via the suppression of nuclear factor kappa-B (NF-κB) activity and expression of MMP-9 and by inhibiting mitogen-activated protein kinase (MAPK) and ERK pathways^11,20,21^. However, in some cancer types, the KISS1/KISS1R pathway has been associated with tumor progression and increased metastatic ability^18^. In fact, in triple-negative breast cancer cells, KISS1R was shown to regulate the transactivation of epidermal growth factor receptor (EGFR or ErbB1) via the actin cytoskeletal binding protein IQGAP1^11,22^. KP-10 enhances this interaction, whereas the KISS1R antagonist P234 inhibits KP-10-induced cell invasion and EGFR activation^11,22^. Moreover, transforming growth factor-β (TGF-β) signaling was reported to induce KISS1 expression and promote triple-negative breast cancer invasion in an ERK/MMP-9-dependent manner^10^. Furthermore, the KISS1/KISS1R pathway has a role in the tight regulation of embryo implantation and trophoblast invasion. Moreover, KP-10 inhibits MMPs and vascular endothelial growth factor (VEGF) expression and stimulates tissue inhibitors of matrix metalloproteinases (TIMPs) in primary trophoblast cells, possibly via ERK1/2 signaling^23^. The effects above might be KISS1R-mediated processes since peptide 356, a KISS1R antagonist, blocked the effects of KP-10^23^.

In contrast, the function of the KISS1R in the cardiovascular system is not well characterized yet. Kisspeptin-10 (KP-10) was reported to have vasoconstrictor^24^, angiogenesis inhibitory^25^, pro-fibrotic^26^, and atherosclerotic effects^27^. Interestingly, the atherosclerotic effects of KP-10 were abolished by the KISS1R antagonist P234 in ApoE^−/−^ mice^27^. However, there is no literature data available on the effects of KISS1R-mediated signaling pathways in uremic cardiomyopathy. Therefore, in our current study, we aimed to investigate whether the KISS1R antagonist P234 can prevent the development of uremic cardiomyopathy in a rat model of CKD induced by 5/6 nephrectomy.

## Results

### P234 administration did not influence the serum and urine markers of CKD

**Figure 1:**
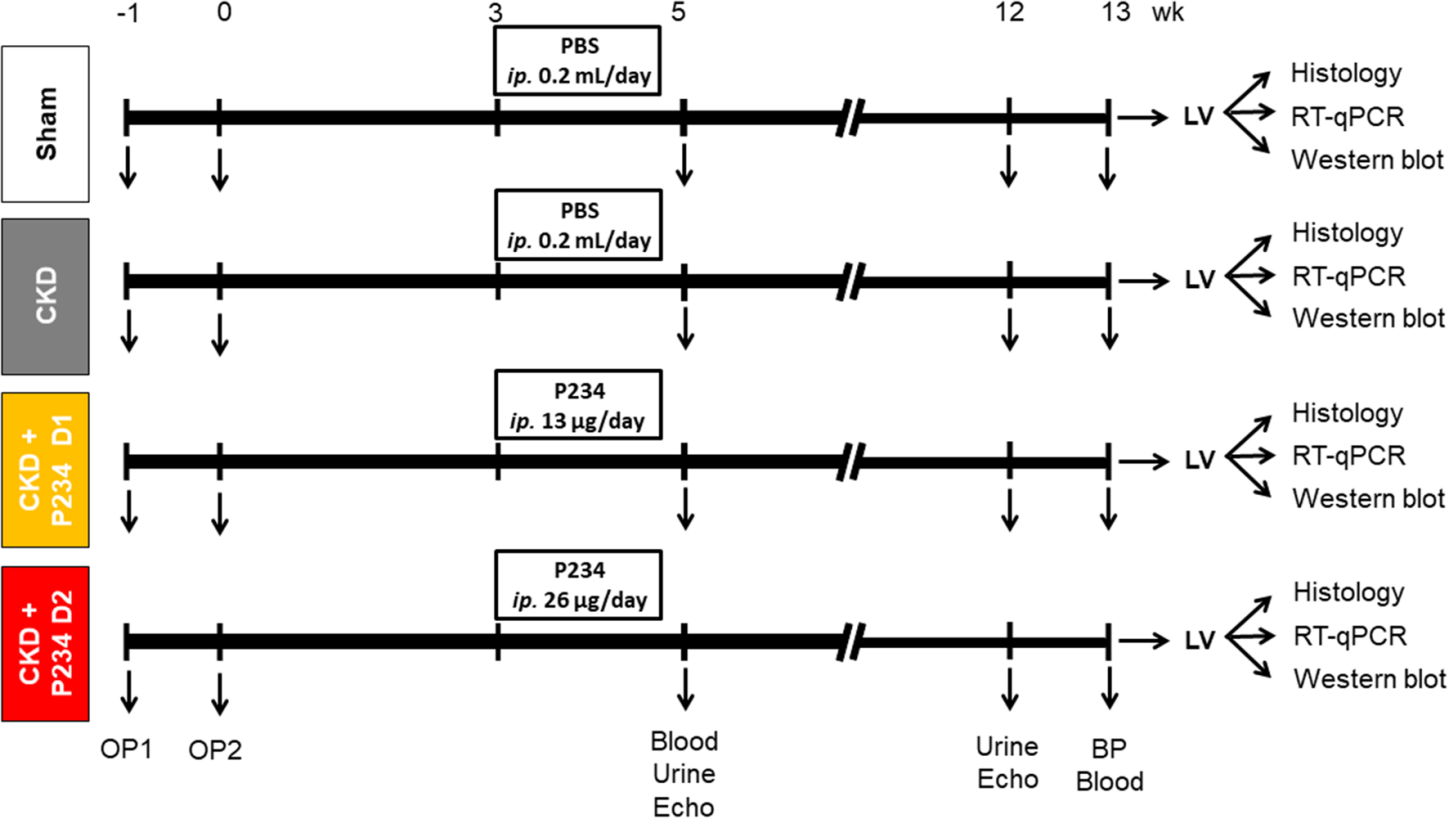
Experiment setup. Blood: blood sampling, BP: blood pressure, Urine: urine sampling, Echo: echocardiography, CKD: chronic kidney disease, PBS: phosphate-buffered saline, P234: KISS1R antagonist peptide-234, LV: left ventricle, Op: operation. Sham: sham-operated group, CKD: chronic kidney disease group, CKD + P234 D1: chronic kidney disease group treated with the lower dose (13 μg/day, dose 1) of KISS1R antagonist peptide-234, CKD + P234 D2: chronic kidney disease group treated with the higher dose (26 μg/day, dose 2) of KISS1R antagonist peptide-234.

**Figure 2:**
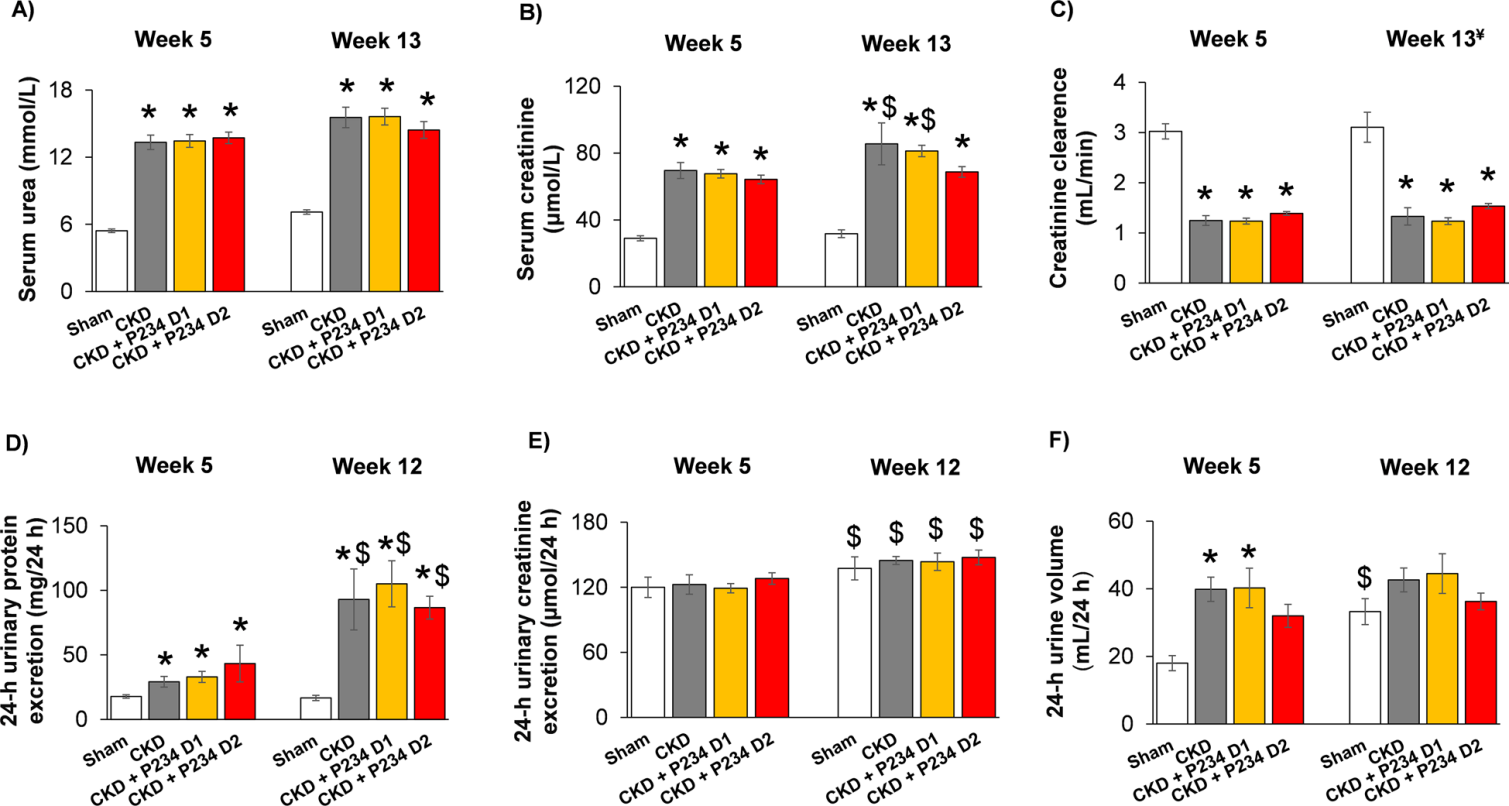
The effects of the KISS1R antagonist peptide-234 on the development of CKD in 5/6 nephrectomized rats. (**A**) Serum urea concentration, (**B**) serum creatinine concentration, (**C**) creatinine clearance, (**D**) 24-h urinary protein excretion, (E) 24-h urinary creatinine excretion, and (F) 24-h urine volume at the endpoint. Values are presented as mean ± S.E.M., *p < 0.05 vs. sham group (n = 7–8, One-Way ANOVA, Holm-Sidak post hoc test), ^$^p < 0.05 vs. the week 5 values in the same group (n = 7–8, Two-Way Repeated Measures ANOVA, Holm-Sidak post hoc test). Creatinine clearance was calculated according to the standard formula (urine creatinine concentration [μM] × urine volume for 24 h [mL])/(serum creatinine concentration [μM] × 24 × 60 min). ^¥^At the endpoint, urine volume and creatinine concentration were measured at week 12 and serum creatinine concentration at week 13. Sham: sham-operated group, CKD: chronic kidney disease group, CKD + P234 D1: chronic kidney disease group treated with the lower dose (13 μg/day, dose 1) of KISS1R antagonist peptide-234, CKD + P234 D2: chronic kidney disease group treated with the higher dose (26 μg/day, dose 2) of KISS1R antagonist peptide-234.

At the 5th and 12th–13th follow-up weeks, serum and urine parameters were determined to verify the development of CKD (Fig. 1). At week 5 and the endpoint, the 5/6 nephrectomized rats showed significantly higher serum urea and creatinine concentrations and decreased creatinine clearance compared to the sham group, indicating the development of CKD regardless of P234 administration (Fig. 2A–C). Notably, serum creatinine levels were markedly higher in the CKD-only group and after the lower dose of P234 treatment at the endpoint compared to the week 5 values in the same groups (Fig. 2B). At week 5 and the endpoint, the 24-h urinary protein excretions were significantly increased in all CKD groups independently of P234 treatment compared to the time-matched sham group (Fig. 2D). At the endpoint, the 24-h urinary protein excretions were markedly increased in all CKD groups compared to the values of the same CKD groups at week 5, indicating worsening glomerular function in CKD (Fig. 2D). At week 5 and the endpoint, there were no significant differences in the 24-h urinary creatinine excretions between the time-matched groups (Fig. 2E). Notably, the 24 h urinary creatinine excretions were markedly higher in all groups at the endpoint compared to the values of the same groups at week 5, probably, due to the growth of the animals (Fig. 2E). At week 5, the 24-h urine volume was significantly higher in the CKD-only and the lower dose of the P234-treated CKD groups compared to the sham group (Fig. 2F). However, the 24-h urine volume showed only a trend toward an increase (p = 0.053) in response to the higher dose of P234 compared to the sham group at week 5 (Fig. 2F). At the endpoint, the 24-h urine volume tended to increase (p = 0.095) in the CKD-only group compared to the sham group, and there was no significant difference between the P234-treated CKD and the sham groups (Fig. 2F).

### The higher dose of P234 administration further increased the serum cholesterol and triglyceride levels in CKD

Selected cardiovascular risk factors, including serum total cholesterol and triglyceride levels at week 13, are reported in Table S1. Serum cholesterol concentrations were markedly higher in all CKD groups compared to the sham group (Table S1). Moreover, it was significantly higher in response to the higher dose of P234 compared to the CKD-only group (Table S1). Serum triglyceride levels showed an increasing tendency (p = 0.129) in the CKD-only group compared to the sham group. It was markedly elevated in response to the higher dose of P234 compared to the sham or CKD-only group (Table S1).

### The P234 administration moderately increased the systolic blood pressure in CKD

Hypertension is a well-known complication and an independent risk factor for developing LVH in CKD. At week 13, the systolic arterial blood pressure was slightly increased (p = 0.170) in the CKD-only group compared to the sham group (Table S1). Interestingly, the systolic blood pressure was significantly increased, and the mean arterial blood pressure showed an increasing tendency (p = 0.07) in the lower dose of the P234-treated CKD group compared to the sham group (Table S1). In the higher dose of the P234-treated CKD group, the systolic blood pressure was tendentiously increased (p = 0.127) compared to the sham group (Table S1). However, there were no significant differences in the diastolic blood pressure values between the groups (Table S1).

### The higher dose of P234 administration increased the diastolic posterior wall thickness at week 5 in CKD

**Figure 3:**
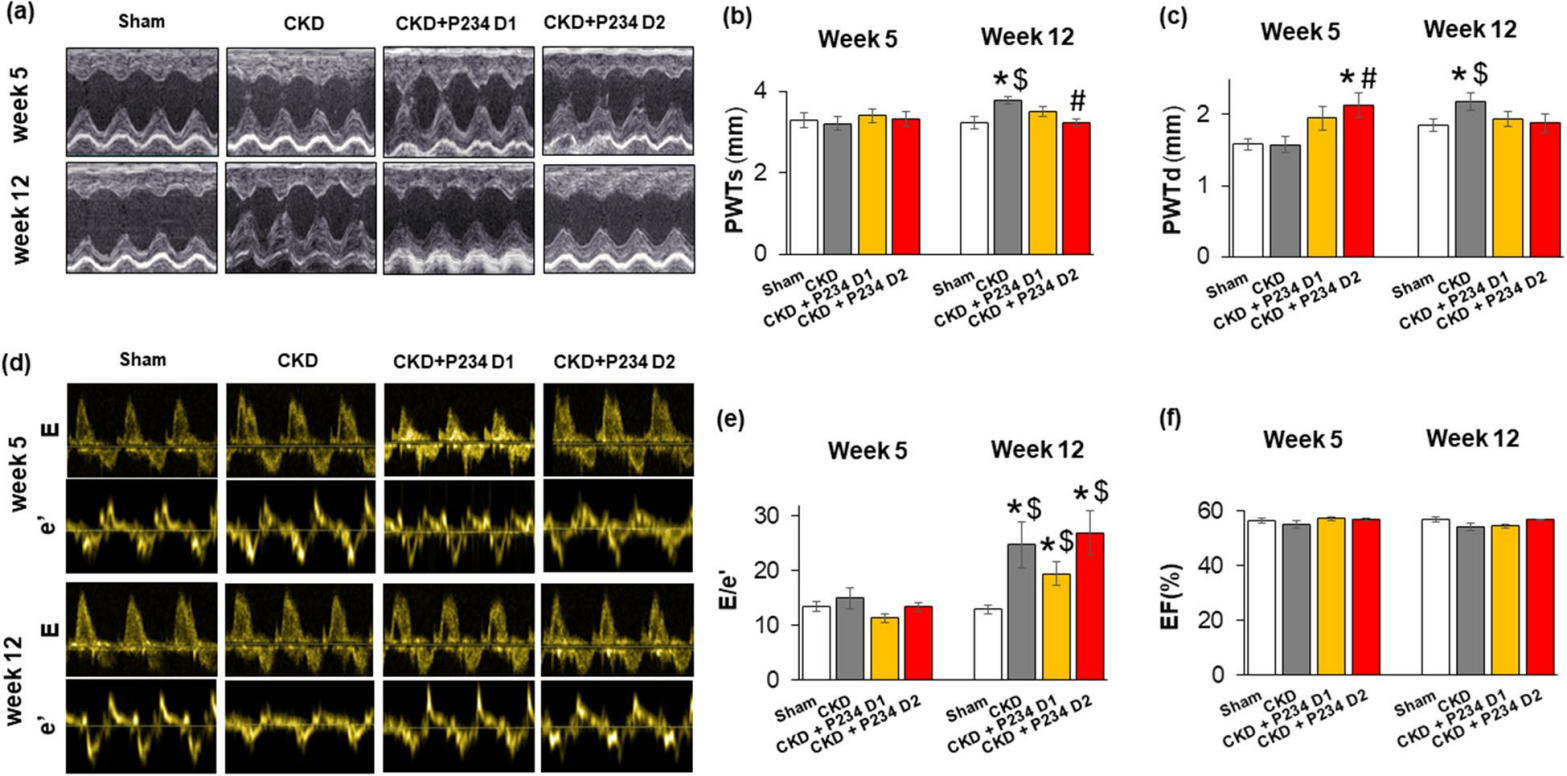
The effects of the KISS1R antagonist peptide-234 on the echocardiographic parameters. (**a**) Representative M-mode images, (**b**) systolic posterior wall thickness (PWTs), (**c**) diastolic posterior wall thickness (PWTd), (**d**) Representative pulse wave and tissue Doppler images of mitral valve early flow velocity (**e**) and septal mitral annulus (e′) velocity, E) E/e′ ratio, (**f**) ejection fraction (EF). Values are presented as mean ± S.E.M., *p < 0.05 vs. sham, ^#^p < 0.05 vs. CKD (n = 7–9, One-Way ANOVA, Holm-Sidak post hoc test). ^$^p < 0.05 vs. week 5 values in the same group (n = 7–8, Two-Way Repeated-Measures ANOVA, Holm-Sidak post hoc test). Sham: sham-operated group, CKD: chronic kidney disease group, CKD + P234 D1: chronic kidney disease group treated with the lower dose (13 μg/day, dose 1) of KISS1R antagonist peptide-234, CKD + P234 D2: chronic kidney disease group treated with the higher dose (26 μg/day, dose 2) of KISS1R antagonist peptide-234.

At week 5, the heart rate was significantly lower in the CKD-only group compared to the sham group (Table 1). There was no significant difference in the other measured echocardiographic parameters between the CKD-only and sham groups (Fig. 3a–f, Table 1). In response to the lower dose of P234, the heart rate remained markedly lower compared to the sham group (Table 1). Notably, the diastolic posterior wall thicknesses tended to increase in the lower dose of the P234-treated group compared to the sham (p = 0.066) or the CKD-only (p = 0.083) groups (Fig. 3, Table 1). In response to the higher dose of P234, the diastolic posterior wall thicknesses were markedly increased, accompanied by a slight thickening of the diastolic septal wall (p = 0.220) compared to the sham or the CKD-only groups (Fig. 3, Table 1), suggesting an earlier LVH development in response to P234 administration in CKD. Notably, the e′ was tendentiously decreased (p = 0.06) in the CKD-only group compared to the sham group, indicating the development of mild diastolic dysfunction in the early phase of CKD. Interestingly, the E velocity was significantly smaller in the lower dose of the P234-treated CKD group compared to the sham group (Table 1). There was no significant difference in the other morphologic and functional parameters measured by echocardiography at week 5 (Table 1).

**Table 1:** The Effects of the KISS1R antagonist peptide-234 on the echocardiographic parameters at weeks 5 and 12.

Parameter (unit)	Wek 5				Week 12			
	Sham	CKD	CKD + P234 D1	CKD + P234 D2	Sham	CKD	CKD + P234 D1	CKD + P234 D2
AWTs (mm)	3.21 ± 0.17	3.37 ± 0.12	3.38 ± 0.12	3.26 ± 0.18	3.20 ± 0.14	3.75 ± 0.09*	3.50 ± 0.09	3.27 ± 0.13^#^
AWTd (mm)	1.86 ± 0.06	1.86 ± 0.10	1.78 ± 0.05	1.81 ± 0.06	1.90 ± 0.04	2.07 ± 0.04*	1.91 ± 0.08	1.99 ± 0.11
SWTs (mm)	3.38 ± 0.1	3.40 ± 0.09	3.48 ± 0.19	3.49 ± 0.19	3.35 ± 0.13	4.05 ± 0.09*^$^	3.62 ± 0.1^#^	3.71 ± 0.15
SWTd (mm)	1.89 ± 0.09	1.91 ± 0.15	1.83 ± 0.11	2.09 ± 0.14	2.01 ± 0.07	2.22 ± 0.11	2.11 ± 0.08	2.09 ± 0.05
LVEDD (mm)	7.19 ± 0.17	7.28 ± 0.17	6.91 ± 0.21	6.56 ± 0.34	7.49 ± 0.18	7.18 ± 0.12	7.35 ± 0.14^$^	7.54 ± 0.24^$^
LVESD (mm)	2.88 ± 0.27	3.02 ± 0.24	2.75 ± 0.26	3.11 ± 0.31	3.58 ± 0.28	2.88 ± 0.27	3.1 ± 0.25	3.54 ± 0.26
FS (%)	60 ± 3	59 ± 3	60 ± 3	53 ± 3	52 ± 3	63 ± 3*	58 ± 3	53 ± 2^#^
EF (%)	92 ± 2	91 ± 2	92 ± 2	87 ± 2	86 ± 3	93 ± 1	91 ± 2	88 ± 2
SV (µL)	108 ± 7	102 ± 8	100 ± 7	105 ± 7	131 ± 6	134 ± 10	132 ± 8	147 ± 6
LVEDV (uL)	191 ± 10	184 ± 12	174 ± 12	184 ± 13	231 ± 8	250 ± 21	243 ± 15	260 ± 12
LVESV (uL)	83 ± 4	82 ± 6	74 ± 5	79 ± 6	100 ± 4	116 ± 11	111 ± 8	113 ± 6
CO (mL/min)	43 ± 3	36 ± 3	36 ± 2	40 ± 3	52 ± 3	50 ± 3	49 ± 3	56 ± 3
HR (1/min)	399 ± 14	358 ± 9*	359 ± 11*	387 ± 9	393 ± 14	371 ± 13	371 ± 13	381 ± 13
E (m/s)	1.02 ± 0.06	1.04 ± 0.11	0.79 ± 0.04*	1 ± 0.06	1.04 ± 0.04	0.99 ± 0.06	0.94 ± 0.05	1.03 ± 0.06
e′ (m/s)	0.076 ± 0.004	0.067 ± 0.008	0.071 ± 0.004	0.075 ± 0.004	0.083 ± 0.006	0.046 ± 0.006*^$^	0.051 ± 0.004*^$^	0.043 ± 0.005*^$^

Values are presented as mean ± S.E.M., *p < 0.05 vs. sham, ^#^p < 0.05 vs. CKD (n = 7–8, one-way ANOVA, Holm-Sidak post hoc test), ^$^p < 0.05 vs. week 5 in the same group (n = 7–8, Two-ways repeated-measures ANOVA, Holm-Sidak post hoc test). Sham: sham-operated group, CKD: chronic kidney disease group, CKD + P234 D1: chronic kidney disease group treated with the lower dose (13 μg/day, dose 1) of KISS1R antagonist peptide-234, CKD + P234 D2: chronic kidney disease group treated with the higher dose (26 μg/day, dose 2) of KISS1R antagonist peptide-234. AWT: anterior wall thickness, CO: cardiac output, d: diastole, E: early ventricular filling velocity, e′: diastolic septal mitral annulus velocity, EF: ejection fraction, FS: fractional shortening, HR: heart rate, LVEDD: left ventricular end-diastolic diameter, LVESD: left ventricular end-systolic diameter, s: systole, SWT: septal wall thickness, SV: stroke volume.

### The higher dose of P234 administration resulted in thinner anterior and posterior walls in CKD at the endpoint

At week 12, the CKD-only group showed a significant increase in the systolic anterior, posterior, and septal wall thicknesses as well as the diastolic anterior and posterior wall thicknesses (Fig. 3a–c, Table 1), indicating the development of a concentric LVH in CKD. The systolic and diastolic posterior and the systolic septal wall thicknesses were significantly thicker in the CKD-only group at week 12 compared to the week 5 values, pointing out the progression of LVH (Fig. 3a–c, Table 1). Consequently, the LV end-systolic and end-diastolic internal diameters showed a trend toward a decrease (p = 0.16 and p = 0.09, respectively), and the fractional shortening was significantly increased in the CKD-only group compared to the sham group (Table 1). There was no significant difference in the ejection fraction between the CKD-only and sham groups measured on the four-chamber view (Fig. 3f) or M-mode images (Table 1). However, diastolic dysfunction developed in the CKD-only group, indicated by the significantly lower e′ (Table 1) and the markedly elevated E/e′ ratio compared to the sham group or the week 5 values in the same group (Table 1, Fig. 3d and e).

In response to the lower dose of P234, the systolic anterior wall thickness showed an increasing tendency (p = 0.087) compared to the sham group (Table 1). Importantly, the systolic septal wall thickness was significantly thinner, and the systolic anterior and posterior wall thicknesses showed a decreasing tendency (p = 0.08 and p = 0.093, respectively) compared to the CKD-only group (Fig. 3a–c, Table 1). In addition, the left ventricular end-diastolic diameter was markedly increased in the lower dose of the P234-treated CKD group at week 12 compared to the week 5 value (Table 1). The fractional shortening and the ejection fraction were unchanged in response to the lower dose of P234 compared to the sham group (Table 1). Interestingly, the E/e′ remained significantly higher (Fig. 3d and e), and the e′ was markedly decreased compared to the sham group or the same group at week 5 (Table 1).

In response to the higher dose of P234, the systolic anterior and posterior wall thicknesses were markedly thinner compared to the CKD-only group (Fig. 3a–c, Table 1). Moreover, the left ventricular end-diastolic diameter was significantly increased at the endpoint compared to the week 5 values (Table 1). Accordingly, the fractional shortening was significantly lower in response to the higher dose of P234 compared to the CKD-only group (Table 1); but the ejection fraction was not significantly different between the groups (Fig. 3f, Table 1). Additionally, the indices of diastolic dysfunction, E/e′ was significantly higher, and the e′ was markedly lower in the higher dose of the P234-treated CKD group compared to the sham group or the same group at week 5 (Fig. 3e, Table 1).

### The higher dose of P234 administration further increased the lung weight in CKD

At week 13, the groups showed no marked difference in body weight, right ventricular (RV) weight, and tibia length (Table S2). The heart weight, left ventricular (LV) weight, and LV weight to tibia length ratio showed a significant increase in all CKD groups compared to the sham group, showing marked LVH (Table S2). The lung weight was significantly higher in all CKD groups, indicating the presence of pulmonary edema (Table S2). Notably, the lung weight showed an increasing tendency in response to the lower dose of P234 (p = 0.06) and a significant increase in response to the higher dose of P234 compared to the CKD-only group, suggesting the development of a more severe HF (Table S2). Additionally, the higher dose of P234 resulted in a significantly higher liver weight compared to the sham group (Table S2). The weight of the remnant left kidney was markedly higher in all CKD groups than the weight of the whole left kidney in the sham group, indicating a frank compensatory renal hypertrophy in CKD (Table S2).

### The higher dose of P234 administration aggravated cardiomyocyte hypertrophy and interstitial fibrosis in CKD

**Figure 4:**
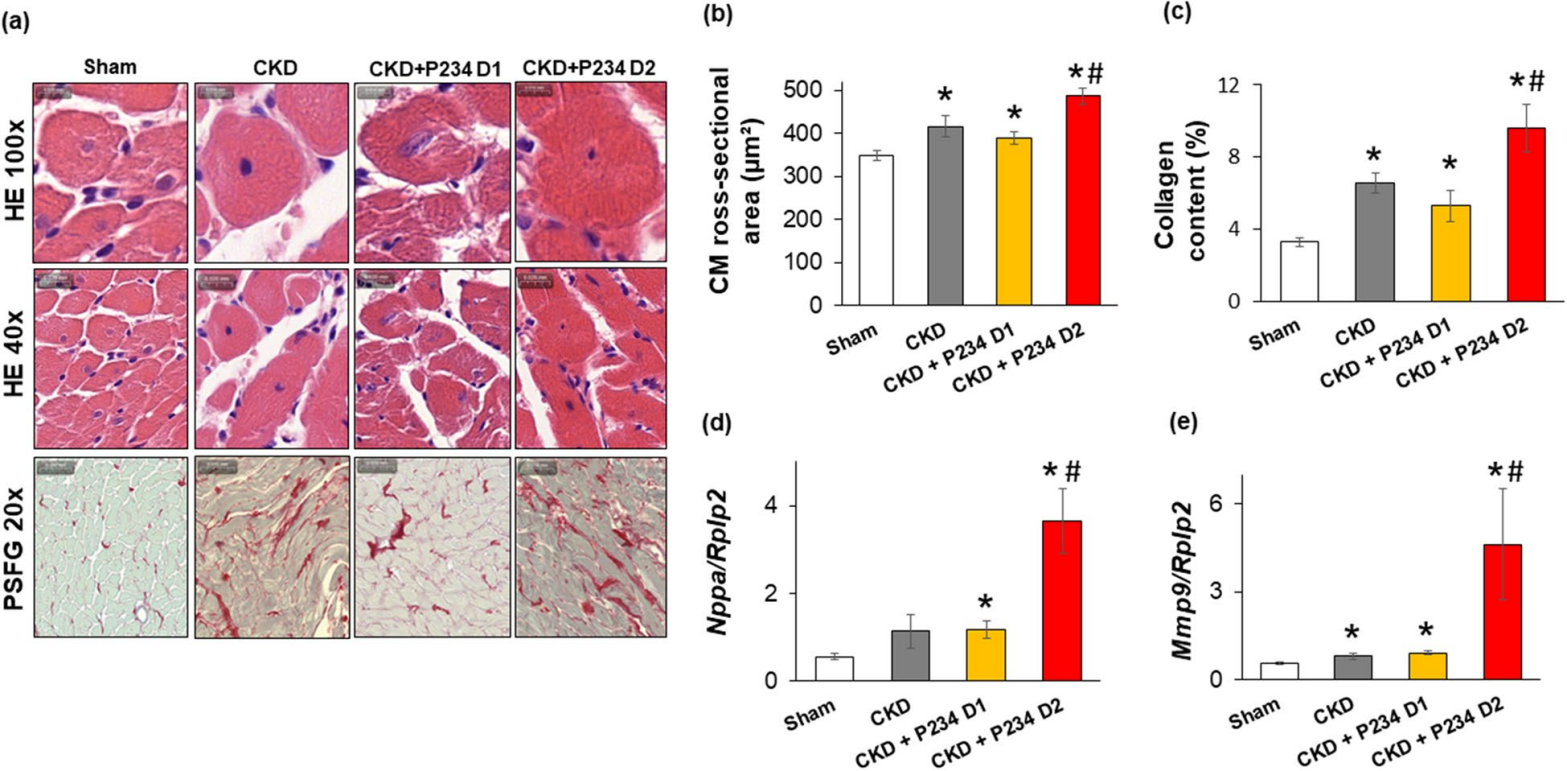
The effects of the KISS1R antagonist peptide-234 on cardiomyocyte hypertrophy and interstitial fibrosis and molecular markers of heart failure at week 13. (**a**) Representative hematoxylin–eosin (HE)-stained slides at 100 × and 40 × magnifications and representative picrosirius red and fast green (PSFG)-stained slides at 20 × magnifcation (**b**) cardiomyocyte cross-sectional areas, (**c**) left ventricular collagen content, (**d**) A-type natriuretic peptide (*Nppa*), and (E) matrix metalloproteinase-9 (*Mmp9*) expressions in the left ventricles normalized to the ribosomal protein lateral stalk subunit P2 (*Rplp2*) gene expression. On the digital HE images, cardiomyocyte cross-sectional areas were measured in 100 selected cardiomyocytes on left ventricular sections cut on the same plane. The mean values of the collagen content of 10 representative PSFG-stained images were calculated and used for statistical evaluation in the case of each left ventricular slide. Scale bars represent 10 µm at the 100 × magnifed images, 20 µm at the 40 × magnifed images, and 50 µm at the 20 × magnifed images. Values are presented as mean ± S.E.M., *p < 0.05 vs. sham, ^#^p < 0.05 vs. CKD (n = 7–8, one-way ANOVA, Holm-Sidak post hoc test). Sham: sham-operated group, CKD: chronic kidney disease group, CKD + P234 D1: chronic kidney disease group treated with the lower dose (13 μg/day, dose 1) of KISS1R antagonist peptide-234, CKD + P234 D2: chronic kidney disease group treated with the higher dose (26 μg/day, dose 2) of KISS1R antagonist peptide-234.

Cardiomyocyte diameters, perimeters, and cross-sectional areas were measured on hematoxyline-eosin (HE)-stained histological slides, and interstitial collagen content was measured on picrosirius red and fast green (PSFG)-stained slides to verify the development of LVH and fibrosis, respectively (Figs. 4a–c, S1). Cardiomyocytes showed significantly enlarged diameters, perimeters, and cross-sectional areas in all CKD groups, confirming the development of LVH at the cellular level (Fig. 4a and b). Moreover, the higher dose of P234 further increased the cardiomyocyte diameter, perimeter, and cross-sectional area compared to the CKD-only group (Figs. S1, 4a and b,). Significantly higher interstitial collagen content was found in all CKD groups compared to the sham group (Fig. 4c). Moreover, the higher dose of P234 further increased the interstitial collagen content compared to the CKD-only group, aggravating the fibrosis and supporting the echocardiographic results at the endpoint (Fig. 4c). Additionally, the expression of atrial natriuretic peptide (*Nppa*) in the LV, a marker of myocardial stretch, showed an increasing tendency (p = 0.083), and the collagenase matrix metalloprotease-9 (*Mmp9*) was significantly overexpressed in the CKD-only group compared to the sham group (Fig. 4d and e). The expressions of *Nppa* and *Mmp9* were significantly increased in response to both doses of P234 compared to the sham group (Fig. 4d and e). In response to the higher dose of P234, *Nppa* and *Mmp9* were further overexpressed compared to the CKD-only group (Fig. 4d and e).

To further investigate the potential fibrotic effect of P234, we measured the expressions of *Col1a1, Mmp9,* and alpha-smooth muscle actin (*Acta2*) in human ventricular cardiac fibroblasts (HVCFs) exposed to TGF-β in the presence or absence of P234 (Fig. S2). TGF-β and P234 exposures were accompanied by slightly elevated transcript levels of *Col1a1* (p = 0.159 and p = 0.135, respectively), whereas *Mmp9* and *Acta2* expressions showed no significant difference between the groups in HVCFs (Fig. S2)*.* Interestingly, the co-treatment of HVCFs with TGF-β and P234 led to significant overexpression of *Mmp9* compared to the control group (Fig. S2).

### The higher dose of P234 induced overexpression of cardiac fibrosis markers in CKD

**Figure 5:**
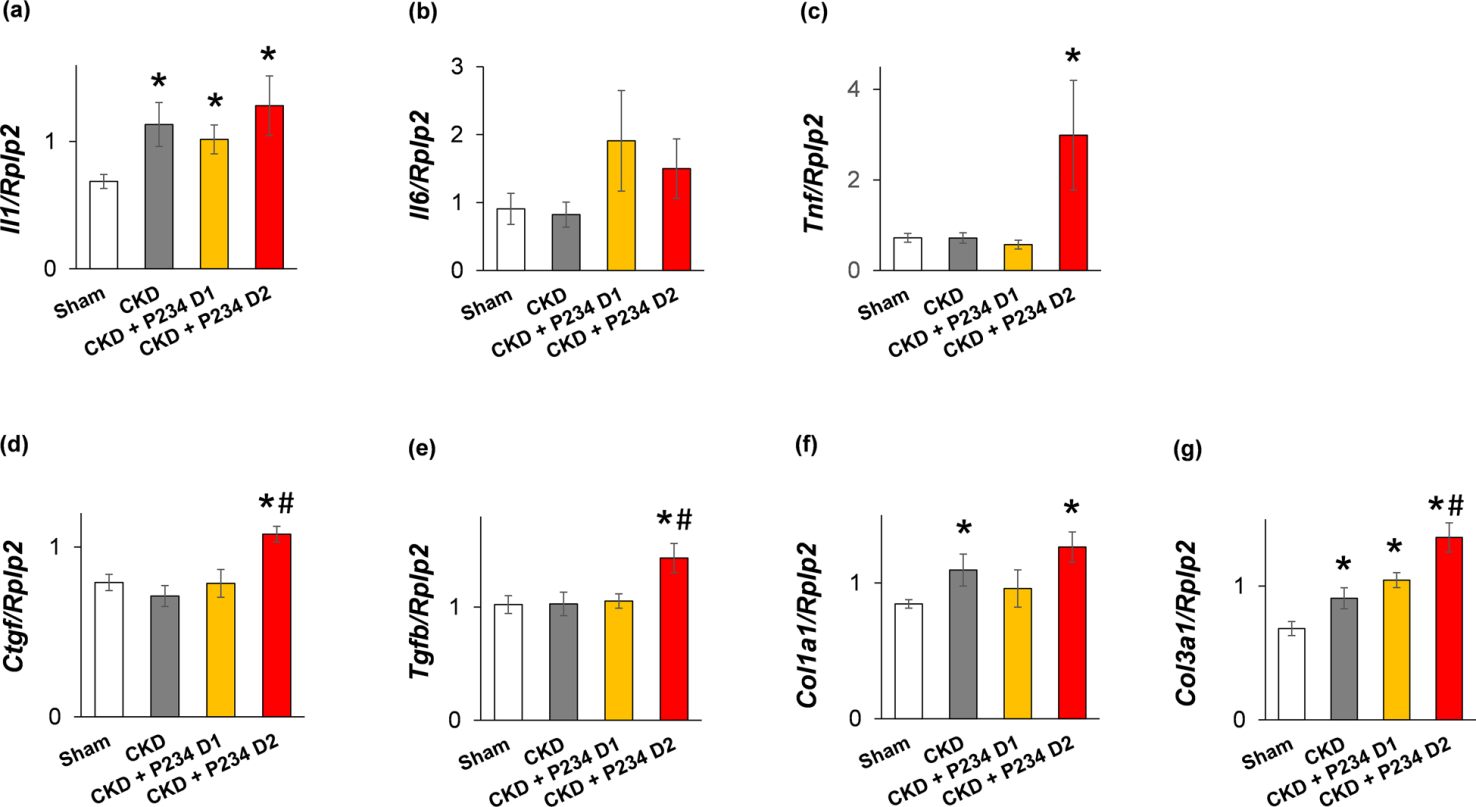
The effects of the KISS1R antagonist peptide-234 on the left ventricular expression of genes associated with inflammation and fibrosis. Relative gene expression of (**a**) interleukin-1 (*Il1*), (**b**), interleukin-6 (*Il6*), (**c**) tumor necrosis factor-α (*Tnf*), (**d**) connective tissue growth factor (*Ctgf*), (**e**) transforming growth factor-β (*Tgfb*), (**f**) collagen type 1 alpha 1 chain (*Col1a1*) and (**g**), collagen type 3 alpha 1 chain (*Col3a1*) normalized to the ribosomal protein lateral stalk subunit P2 (*Rplp2*) gene expression. Values are presented as mean ± S.E.M., *p < 0.05 vs. sham, ^#^p < 0.05 vs. CKD (n = 7–8, One-Way ANOVA, Holm-Sidak post hoc test). Sham: sham-operated group, CKD: chronic kidney disease group, CKD + P234 D1: chronic kidney disease group treated with the lower dose (13 μg/day, dose 1) of KISS1R antagonist peptide-234, CKD + P234 D2: chronic kidney disease group treated with the higher dose (26 μg/day, dose 2) of KISS1R antagonist peptide-234.

To underpin our results on fibrosis, we evaluated the LV expression of inflammatory and fibrosis markers using RT-qPCR (Fig. 5a–g). Among the investigated inflammatory markers, interleukin-1 (*Il1*) was significantly overexpressed in all CKD groups compared to the sham group (Fig. 5a). There was no significant difference in the LV expressions of interleukin-6 (*Il6*) and tumor necrosis factor-α (*Tnf*) between the CKD-only and sham groups (Fig. 5b and c). Notably, *Il6* expression showed an increasing tendency in response to both doses of P234 (p = 0.175 and p = 0.110, respectively) compared to the sham group (Fig. 5b). Additionally, the higher dose of P234 resulted in a significant increase in the *Tnf* expression compared to the sham group (Fig. 5c). There were no significant differences between the connective tissue growth factor (*Ctgf*) and transforming growth factor-β (*Tgfb*) expressions between the CKD-only and sham groups (Fig. 5d and e). Collagen-1a1 (*Col1a1*) and collagen-3 (*Col3a1*) were overexpressed in the CKD-only group, indicating an active fibrotic process in CKD (Fig. 5f and g). The *Ctgf, Tgfb,* and *Col1a1* expressions failed to increase in response to the lower dose of P234 compared to the CKD-only or sham groups (Fig. 5d–f). The *Col3a1* was overexpressed in response to the lower dose of P234 compared to the sham group (Fig. 5g). In contrast, the higher dose of P234 resulted in a significantly higher expression of *Ctgf, Tgfb*, and *Col3a1* compared to the CKD-only group, whereas the *Col1a1* expression was markedly upregulated compared to the sham group (Fig. 5d–g).

### P234 administration resulted in elevated left ventricular pERK1/ERK1 ratio in CKD

**Figure 6:**
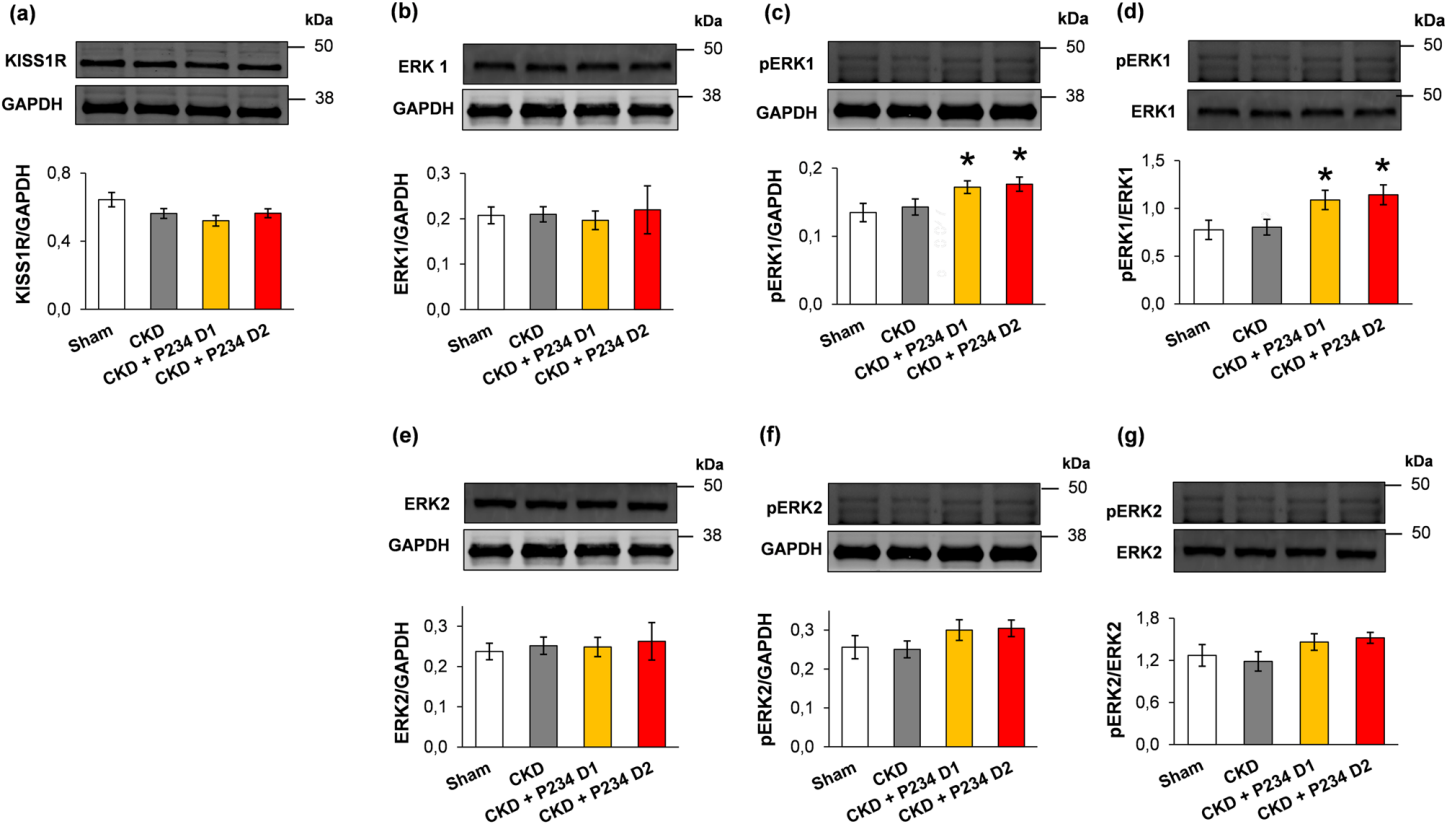
The effects of the KISS1R antagonist peptide-234 on the protein levels of KISS1R and ERK1/2 at week 13. Left ventricular protein levels and cropped representative Western blot imagines of (**a**) Kisspeptin receptor-1 (KISS1R, 40–140 kDa), (**b**) total ERK1 (44 kDa), (**c**) phospho-ERK1 (pERK1, 44 kDa), (**d**) pERK1/ERK1 ratio and (**e**) total ERK 2 (42 kDa), (**f**) phospho-ERK2 (pERK2, 42 kDa), (**g**) pERK2/ERK2 ratio. Values are presented as mean ± S.E.M., *p < 0.05 vs. sham (n = 7, One-Way ANOVA, Holm-Sidak post hoc test). Sham: sham-operated group, CKD: chronic kidney disease group, CKD + P234 D1: chronic kidney disease group treated with the lower dose (13 μg/day, dose 1) of KISS1R antagonist peptide-234, CKD + P234 D2: chronic kidney disease group treated with the higher dose (26 μg/day, dose 2) of KISS1R antagonist peptide-234. Images were captured with the Odyssey CLx machine and exported with Image Studio 5.2.5 software. The full-length Ponceau-stained membranes and the corresponding Western blot images are presented in the Supplementary Material (Figs. S3–S5).

It was reported in tumor cells that the activation of KISS1R could inhibit ERK1/2, which might subsequently lead to the inhibition of the inflammatory transcription factor NF-κB and the collagenase MMP-9^28^. In our present study, KISS1R protein levels were not significantly different between the groups; however, all CKD groups showed a decreasing tendency in KISS1R protein levels compared to the sham group (Fig. 6a). There was no significant difference in the ERK1, ERK2, pERK1, and pERK2 levels and pERK1/ERK1 and pERK2/ERK2 ratios between the sham and CKD groups (Fig. 6b–g). In response to both doses of P234, the pERK1 level and pERK1/ERK1 ratio were significantly increased compared to the sham group (Fig. 6c and d). In response to the higher dose of P234, pERK2 and pERK2/ERK2 ratio showed a trend to increase (p = 0.10 and p = 0.056, respectively) compared to the sham group (Fig. 6f and g).

### The higher dose of P234 administration resulted in elevated left ventricular expression of apoptosis-associated transcripts in CKD, but the protein levels remained unchanged

**Figure 7:**
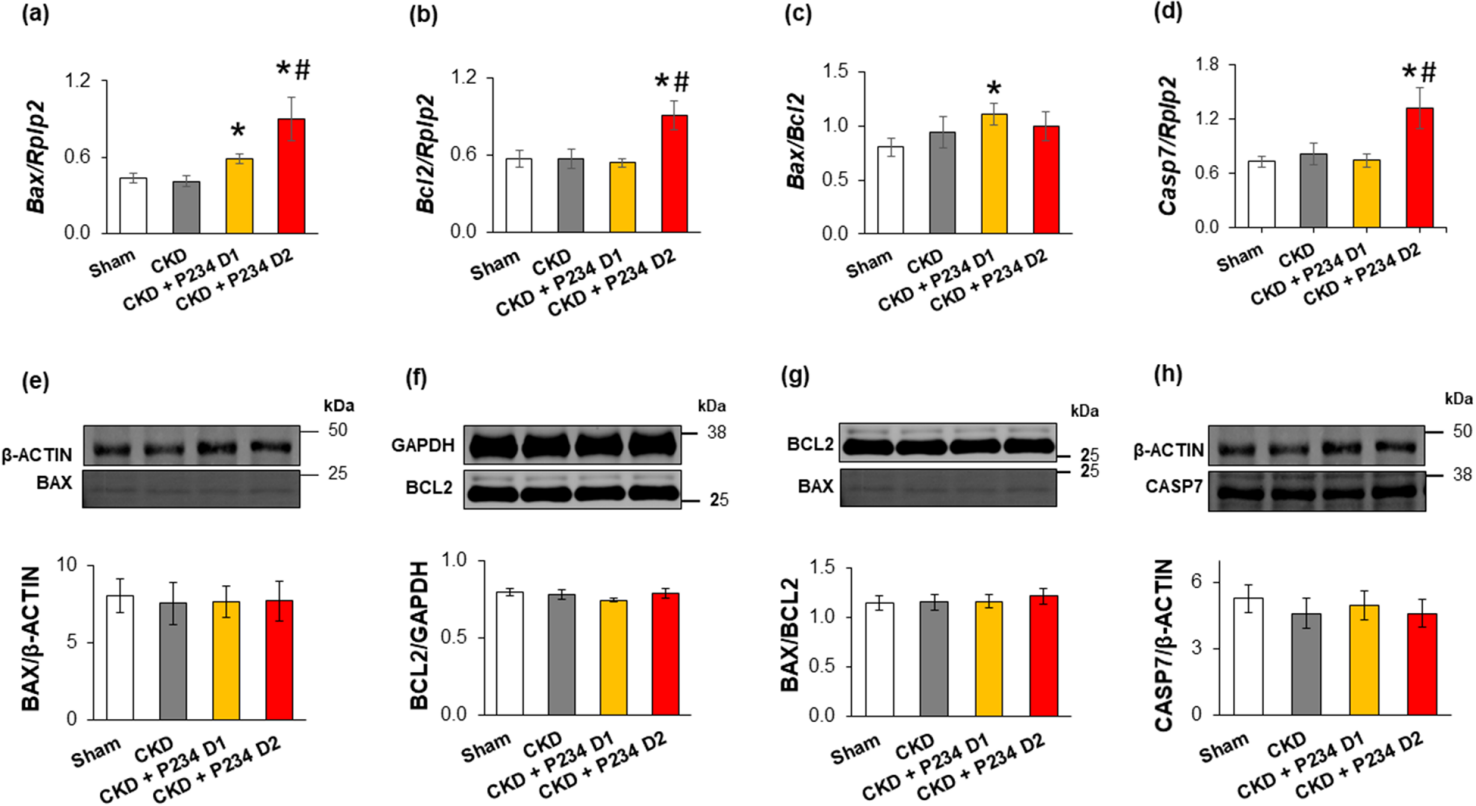
The effects of the KISS1R antagonist peptide-234 on apoptosis-associated gene expressions and protein levels in the left ventricles at week 13. Relative gene expression of (**a**) BCL2-associated X apoptosis regulator (*Bax*), (**b**) B-Cell CLL/lymphoma 2 apoptosis regulator (*Bcl2*), (**c**) *Bax/Bcl2* ratio, and (**d**) caspase7 (*Casp7*) normalized to the ribosomal protein lateral stalk subunit P2 (*Rplp2*) gene expression. Left ventricular protein levels and cropped representative imagines of (**e**) BAX (20 kDa), (**f**) BCL2 (26 kDa), (**g**) BAX/BCL2 ratio, and (**h**) CASP 7 (35 kDa). Values are presented as mean ± S.E.M., *p < 0.05 vs. sham, ^#^p < 0.05 vs. CKD vehicle group (n = 7–8 for RT-qPCR and n = 7 for Western blot measurements, One-Way ANOVA, Holm-Sidak post hoc test). Sham: sham-operated group, CKD: chronic kidney disease group, CKD + P234 D1: chronic kidney disease group treated with the lower dose (13 μg/day, dose 1) of KISS1R antagonist peptide-234, CKD + P234 D2: chronic kidney disease group treated with the higher dose (26 μg/day, dose 2) of KISS1R antagonist peptide-234. Images were captured with the Odyssey CLx machine and exported with Image Studio 5.2.5 software. The full-length Ponceau-stained membranes and the corresponding Western blot images are presented in the Supplementary Material (Figs. S6–S8).

There was no significant difference in the LV expression of selected apoptosis-associated markers (Bcl-2 Associated X-protein [*Bax*]*,* B-Cell CLL/lymphoma-2 apoptosis regulator [*Bcl2*], *Bax/Bcl2* ratio, and caspase-7 [*Casp7*]) between the CKD-only and sham groups (Fig. 7a–d). In contrast, the *Bax* expression and the *Bax/Bcl2* ratio were significantly increased in response to the lower dose of P234 compared to the sham group (Fig. 7a and c). Moreover, the higher dose of P234 led to increased expressions of *Bax, Bcl2,* and *Casp7* compared to the CKD-only group (Fig. 7a, b, and d). Interestingly, there were no significant differences in the BAX and BCL2 protein levels, the BAX/BCL2 ratio, and CASP7 protein levels between the groups (Fig. 7e–h).

## Discussion

The present study demonstrates for the first time that the KISS1R antagonist P234 accelerates the progression of uremic cardiomyopathy without worsening the renal function in our rat model of CKD. To investigate the effects of P234 on the development of uremic cardiomyopathy and CKD, the antagonist was administered in an early and sensitive phase of disease development, i.e., 2 weeks after CKD induction. The higher dose of P234 administration was associated with earlier development of LVH at week 5. At the endpoint, the higher dose of P234 administration led to reduced anterior and posterior wall thicknesses with more severe cardiac fibrosis and left ventricular overexpression of several marker genes associated with cardiac remodeling (*Ctgf, Tgfb, Col3a1, Mmp9*) and myocardial stretch (*Nppa*) compared to the CKD-only group. In response to the higher dose of P234, the apoptosis-associated markers (*Bax, Bcl2, Casp7*) were overexpressed only at the mRNA but not at the protein level compared to the CKD-only group. The phospho-ERK1/ERK1 ratio was increased in both P234-treated CKD groups compared to the sham group. The deteriorating effects of the higher dose of P234 in uremic cardiomyopathy were probably associated with the activation of TGF-β-mediated hypertrophic and fibrotic pathways.

KISS1R was previously shown to be expressed in the heart, coronary arteries, aorta, microvasculature, and kidney tissues^29,30^. Our results underlie these observations since we also detected KISS1R in the LV; however, in the CKD groups, the expression of KISS1R was slightly decreased. Similarly, Shoji et al*.* found that KISS1R protein levels were significantly lower in the remnant kidneys of 5/6-nephrectomized rats compared to the sham animals 8 weeks after the operations^31^. In addition, the deletion of KISS1R has been linked to detrimental effects in kidney development, such as the retardation of kidney branching morphogenesis and glomerular development in murine embryos^32^. In general, renal function is often impaired in chronic HF patients, and conversely, HF aggravates renal failure in cardio-renal syndromes^6^. Dysfunction of each organ can induce and perpetuate injury in the other via complex hemodynamic, neurohormonal, and biochemical pathways^5,6,8^. Administration of P234 did not further worsen the routine serum and urine laboratory parameters of renal dysfunction in our rat model of CKD induced by 5/6 nephrectomy. Due to the severity of our CKD model, the possible adverse effects of P234 on kidney function are hard to evaluate. A detailed characterization of renal function and morphology would be necessary to decide whether the deterioration of kidney morphology and/or function plays a role in the worsening of uremic cardiomyopathy in response to P234 in our current model; however, it was out of the scope of our present descriptive study focusing on the heart.

At the endpoint, the severity of CKD in our current rat model corresponds to human G2 or G3a stages with mildly or moderately diminished kidney function based on our present laboratory and echocardiographic findings^33,34^. In accordance with our previous results and other studies, CKD animals developed LVH and diastolic dysfunction after 12 weeks^35–39^, albeit these symptoms were not detectable by echocardiography up to 5 weeks after 5/6 nephrectomy^36,40^. However, characteristic laboratory markers of CKD, including elevated serum urea and creatinine concentrations, urine protein levels, and decreased creatinine clearance, were present at week 5. These findings suggest that renal dysfunction is already started to develop during the early phase of CKD, which is comparable to the stage when CKD can be diagnosed in patients using routine laboratory screening tests. Therefore, we decided to start the administration of P234 in this relatively early phase of CKD, in which manifested LVH, diastolic dysfunction, and concomitant fibrosis is not yet present, to investigate its potential effects on the development of uremic cardiomyopathy.

However, no previous data were published on the possible dosage of the KISS1R antagonist P234 in connection to fibrotic processes or uremic cardiomyopathy. The primary function of kisspeptin is thought to be the regulation of the hypothalamic–pituitary–gonadal axis; therefore, the effects of the different forms of kisspeptin were mainly investigated in connection with reproductive function. In rats, the administration of kisspeptin in a dose range of 7.5–300 nmol intraperitoneally was investigated and found to stimulate gonadotropin-releasing hormone and, consequently, luteinizing hormone^41^. We also have previously investigated the effects of kisspeptin analogs on anxiety in rats and found that a dose of 1–2 μg intracerebroventricularly can exert an anxiety-like behavior in Wistar rats^42,43^. Unfortunately, the literature data on the effects of KISS1R agonists on fibrosis are limited and controversial. In fact, Zhang et al. reported that the KISS1R agonist KP-10 (*sc.* 40 nmol KP-10 dissolved in 200 μL saline for 7 days) led to cardiac interstitial fibrosis and altered the morphology and structure of myocardial cells, serum metabolite levels, and expression of genes and proteins in the heart tissue obtained from healthy male Sprague–Dawley rats^25^. In contrast, Lei et al. showed that another KISS1R agonist, the KP-13 reduced bleomycin-induced pulmonary fibrosis by repressing *Tnf*, *Tgfb*, and *Col1a1*^44^. Therefore, in our present study, we applied doses of the KISS1R antagonist P234 based on the study above on pulmonary fibrosis of Lei et al*.*^43^, in which C57BL/6 mice were administered 1 mg/kg *ip.* KP-13 for 28 days. In our present study, the equimolar dose of P234 in rats was calculated according to the study by Freireich et al*.*^45^. Based on this calculation, we chose 10 nmol (approximately 13 μg, which corresponds to an average of 40 μg/kg/day in a rat of 300–500 g) and a higher dose of 20 nmol (26 μg, corresponding to 80 μg/kg/day) in our present study.

Severe hypertension is usually not a typical feature of CKD in the 5/6 nephrectomy-induced model^39^. Indeed, systolic blood pressure was only moderately increased in our experimental setup. Therefore, we suggest that the factors involved in the development of diastolic dysfunction, LVH, and fibrosis may not be primarily linked to arterial hypertension in this animal model of CKD. However, in response to the higher dose of P234, we observed echocardiographic signs of earlier LVH development (i.e., posterior wall thickening) at week 5. Moreover, at the endpoint, thinner LV anterior and posterior walls and more severe fibrosis developed, accompanied by a higher degree of systolic blood pressure elevation in the higher dose of the P234-treated CKD group compared to the CKD-only group. In contrast to our present findings, Sato et al. reported that 4-week administration of P234 (50 μg/kg/hour) or the KISS1R agonist KP-10 (5 and 12.5 μg/kg/h) did not influence the blood pressure in ApoE^−/−^ atherosclerotic mice^27^. In the mentioned study by Sato et al., the P234 administration prevented the atherosclerotic plaque progression and reduced the macrophage infiltration and vascular inflammation in ApoE^−/−^ mice without affecting the serum cholesterol levels^27^. In contrast, the higher dose of P234 increased the serum cholesterol level in CKD in our present study. Notably, Sato et al*.* fed ApoE^−/−^ mice with a high‐cholesterol diet containing 16.5% fat, 1.25% cholesterol, and 0.5% sodium cholate^27^. Therefore, in their study, P234 administration could probably not increase the serum cholesterol levels further.

Our CKD model showed echocardiographic signs of pathologic cardiac remodeling with LVH at week 5, then LV wall thinning and dilatation, inflammation, and fibrosis accompanied by the LV overexpression of *Il1, Col1a1, Col3a1, Mmp9,* and *Nppa* at the endpoint. These molecular findings are consistent with our previous results and other studies on rat models of CKD^35–37,39^. The TGF-β/SMAD pathway plays a crucial role in the development of cardiac fibrosis and pathologic remodeling by inducing pro-fibrotic gene expression, including, *e.g.*, *Ctgf, Col1a1, Col3a1,* and collagenases, such as *Mmp9*^46^. Interestingly, Tian et al. reported that the *Kiss1* gene encoding kisspeptins could be a downstream target of the TGF-β/SMAD signaling pathway in triple-negative breast cancer cells, promoting tumor growth and invasion^47^. In our present study, the higher dose of P234 increased the expression of inflammatory (*Tnf*), fibrotic (*Ctgf, Tgfb,* and *Col3a1*), heart failure (*Nppa*), and cardiac remodeling-associated (*Mmp9*) genes compared to the CKD-only group, supporting our echocardiographic and histology results. In consonance with our present findings, Lei et al*.* found that the KISS1R agonist KP-13 ameliorated the bleomycin-induced pulmonary inflammation and fibrosis in mice, whereas the KISS1R antagonist P234 abolished the antifibrotic effects of KP-13^44^. A recent study by Guzman et al*.* underlies our findings as well since deletion of the KISS1R resulted in increased inflammation (*Il1, Tnf*) and fibrosis (*Tgfb, Mmp2, Col1a2*) markers in a rodent model of non-alcoholic fatty liver disease and steatohepatitis^48^. Furthermore, in the mentioned study by Guzman et al., a KISS1R agonist alleviated these adverse effects^48^.

It is well known that the activation of the ERK1/2 signaling pathway can induce cardiac hypertrophy and fibrosis^49,50^. In our CKD model, there was no significant difference in the ERK1/2 and pERK1/2 levels between the CKD-only and sham groups, similar to our previous findings in our CKD model induced by 5/6 nephrectomy^35^. A plausible explanation for our findings on ERKs may be that our CKD model represents the compensated phase of LVH from the viewpoint of global LV function (i.e., ejection fraction and fractional shortening). In contrast, P234 seems to activate the ERK1/2 signaling pathway, which might be responsible for the more severe LVH at week 5 and fibrosis at week 13 in CKD.

Pathological cardiac hypertrophy is typically associated with cardiomyocyte apoptosis when the decompensation in cardiac function occurs, and the compensated cardiac hypertrophy (i.e., HFpEF) converts to HFrEF^10^. In our present study, the thicknesses of the systolic posterior and anterior walls were significantly reduced in the higher dose of the P234-treated CKD group compared to the CKD-only group. Moreover, the significantly increased interstitial collagen content and the overexpression of the fibrosis- (*Ctgf*, *Tgfb, Col3a1)* and cardiac remodeling- *(Mmp9)* associated markers suggest a more active remodeling process with fibrosis in the higher dose of P234-treated CKD group compared to the CKD-only group. Interstitial fibrosis can promote tissue hypoxia, which in turn leads to myocardial cell death forms, particularly apoptosis^4^. In our hands, the higher dose of P234 already increased the expression of the apoptotic markers (i.e., *Bax, Casp7,* and *Bax/Bcl2* ratio) at the transcript but not at the protein level at this phase of uremic cardiomyopathy at week 13. Additionally, the re-expression of A-type natriuretic peptide (*Nppa*) in heart failure was associated with increased apoptotic index in hypertrophied ventricular cardiomyocytes^51^. In the current study, the HF marker *Nppa* and the apoptosis markers *Bax* and *Casp7* showed a marked LV overexpression in response to the higher dose of P234 compared to the CKD-only group. This finding is consistent with our present echocardiographic and histology results, indicating the worsening of uremic cardiomyopathy in response to the higher dose of P234.

Like all experimental approaches aiming to recapture the clinical symptoms of CKD, our study owns several limitations. We intended to test the potential cardiac effects of P234 in a rat model of uremic cardiomyopathy. Significant differences exist in the pathomechanisms of experimental and clinical CKD and uremic cardiomyopathy, comprising the used juvenile inbred rat species, the absence of atherosclerosis, diabetes mellitus, less pronounced hypertension, and earlier development of heart failure. Notably, the female sex could slow down the progression and severity of CKD and uremic cardiomyopathy due to female sex hormonal effects^2,30,42^. Therefore, only male rats were used in our present study. Animal models of both sexes with concomitant comorbidities and advanced aging would be more suitable to mirror the clinical scenario of CKD in future studies. Moreover, many unknown mechanisms exist in the development of CKD and uremic cardiomyopathy. We focused primarily on the effects of the KISS1R antagonist P234, but ultimately, further investigations of the effects of KISS1R agonists/KPs on uremic cardiomyopathy and CKD will be warranted. Furthermore, the detailed investigation of cell death and inflammatory mechanisms was out of the scope of the present exploratory study. In future experiments, more doses and therapeutic regimens of P234 should also be tested in more in vitro experiments and in vivo models of CKD.

## Conclusions

The KISS1R antagonist P234 worsened the outcomes of uremic cardiomyopathy in our rat model of CKD, probably, by activating the hypertrophic and fibrotic TGF-β-mediated pathways. The KISS1/KISS1R-mediated pathways might play a role in the development of uremic cardiomyopathy and represent novel drug targets to minimize its detrimental effects.

## Materials and methods

### Ethics declarations

This investigation conformed to the EU Directive 2010/63/EU and the National Institutes of Health Guide for the Care and Use of Laboratory Animals (NIH Publication No. 85-23, revised 1996) and. It was approved by the regional Animal Research Ethics Committee of Csongrád County (XV.2598/2020, date of approval: 18 September 2020) and the University of Szeged in Hungary. All national and institutional guidelines for the care and use of laboratory animals were followed. The authors complied with the ARRIVE guidelines. Consent to participate/consent to participate statements are not applicable.

### Animals

In the present study, 36 male 8 weeks old Wistar rats (*Rattus norvegicus*, 300–350 g) were used. The rats were housed in pairs in individually ventilated cages (Tecniplast Sealsafe IVC system, Buguggiate, Italy) in a temperature-controlled room (22 ± 2 °C; relative humidity 55 ± 10%, 12 h:12 h light/dark cycle). Tap water and standard rat chow were supplied ad libitum.

### Experimental setup

Rats (n = 28) received 5/6 nephrectomy to induce experimental CKD. Control animals underwent a sham operation (n = 8). After the operations, rats were followed up for 13 weeks (Fig. 1). On the first day of the 3rd follow-up week, rats were divided into four groups and treated for 10 days as follows: (i) Vehicle-treated (PBS, *ip.* 0.2 mL/day) sham-operated group (Sham, n = 8), (ii) Vehicle-treated (PBS, *ip.* 0.2 mL/day) CKD group (n = 10), (iii) CKD group treated with a lower dose of P234 (*ip.* 13 µg/day [i.e., 10 nmol/day] dissolved in 0.2 mL PBS, CKD + D1, n = 9), and (iv) CKD group treated with a higher dose of P234 treated (26 µg/day [i.e., 20 nmol/day] dissolved in 0.2 mL PBS, CKD + D2, n = 9). The time course and doses of P234 (Tocris Bioscience, Bristol, UK, catalog No.: 3881, molecular weight: 1295.42 g/mol; peptide sequence: Ac-D-Ala-Asn-Trp-Asn-Gly-Phe-Gly-D-Trp-Arg-Phe-NH_2_) were selected based on our preliminary data and previous studies^26,44^. It is important to emphasize that the KISS1R antagonist P234 was administered in an early and sensitive phase of CKD and uremic cardiomyopathy development, i.e., from the 3rd week after CKD induction before the development of cardiac hypertrophy and diastolic dysfunction. Thus, we aimed to test whether P234 was able to attenuate rather than treat the development of uremic cardiomyopathy.

In our study, 4 animals died postoperatively (2 in the CKD group, 1 in the lower dose of the P234-treated CKD group, and 1 in the higher dose of the P234-treated CKD group). At weeks 5 and 12, cardiac morphology and function were assessed using transthoracic echocardiography (Fig. 1). Blood was collected from the saphenous vein at week 5 and from the abdominal aorta at week 13 to measure serum parameters. The animals were placed into metabolic cages for 24 h at weeks 5 and 12 to measure urine creatinine and protein levels (Fig. 1). At week 13, blood pressure measurements were performed in a subgroup of animals (Fig. 1). At week 13, hearts were isolated, then left ventricular samples were prepared for histology and biochemical measurements. In the CKD groups, the development of LVH and fibrosis were verified by the measurement of cross-sectional areas on hematoxylin–eosin (HE)-stained and picrosirius red/fast green-stained (PSFG) slides, respectively. The expressions of selected marker genes associated with LVH, fibrosis, cardiac remodeling, myocardial stretch, inflammation, and apoptosis were measured by RT-qPCR and/or Western blot in LV tissue samples (Fig. 1). Cell culture experiments partially mimicking in vivo conditions were performed using human ventricular cardiac fibroblasts (HVCFs, see below).

### Subtotal (5/6) nephrectomy model

Subtotal (5/6) nephrectomy and sham operation were performed in two phases (Fig. 1), as described previously^35,36,52^. Before the operations, anesthesia was induced by sodium pentobarbital (Euthasol; 40 mg/kg *ip*.; Produlab Pharma b.v., Raamsdonksveer, The Netherlands). First, the 1/3 left kidney on both poles was excised, and one week later, the right kidney was removed, as described previously^35–37,52^. During the sham operations, only the renal capsules were removed^35–37,52^. After the surgeries, the incision was closed with continuous sutures, and povidone-iodine was applied to the skin’s surface^33^. As a postoperative medication, nalbuphine hydrochloride (0.3 mg/kg *sc.*, Nalbuphine 10 mg/mL, Teva Pharmaceuticals Ltd., Debrecen, Hungary) was administered for 4 days: twice on the first two, then once on the third and fourth postoperative days^33^. Enrofloxacin antibiotics (Enroxil 75 mg tablets, Krka, Novo Mesto, Slovenia; dissolved in tap water in 3.5 mg/L end concentration) were administered in the drinking water for 4 days after the surgeries^33^.

### Transthoracic echocardiography

Left ventricular morphology and function were assessed by transthoracic echocardiography at weeks 5, and 12, as described previously^36,37^ (Fig. 1). Briefly, rats were anesthetized with 2% isoflurane (Forane, AESICA, Queenborough Limited Kent, UK). Then the chest was shaved, and the animals were placed supine on a heating pad^36,37,52^. 2D, M-mode, Doppler, and tissue Doppler echocardiographic examinations were performed according to the criteria of the European Society of Cardiology^53^ on a Vivid IQ ultrasound machine (General Electric Medical Systems, New York, NY, USA) with a phased array transducer of 5.0–11 MHz (12S-RS probe, General Electric Medical Systems, New York, NY, USA)^36,37,54^. Data from three consecutive heart cycles were analyzed by an experienced investigator blinded to the treatment assignment (EchoPac Dimension v201 software; General Electric Medical Systems, New York, NY, USA)^36,37,54^. The mean values of three measurements were calculated and used for statistical analysis^37,54,55^.

### Blood pressure measurement

At week 13, a PE50 polyethylene catheter (Cole-Parmer, Vernon Hills, IL, USA) was inserted into the left femoral artery under sodium pentobarbital anesthesia (Euthasol; 40 mg/kg; Produlab Pharma b.v., Raamsdonksveer, The Netherlands)^36,37^ (Fig. 1). Blood pressure measurements were performed between 09:00 and 14:00 h with an SEN-02 pressure transducer (MDE Ltd., Budapest, Hungary) connected to an EXP-HG-1 amplifier (MDE Ltd., Budapest, Hungary) and WS-DA data acquisition system (MDE Ltd., Budapest, Hungary)^36,37^. The data were analyzed using the S.P.E.L. Advanced Haemosys software (MDE Ltd., Budapest, Hungary)^36^.

### Serum and urine metabolite concentrations

At weeks 5 and 12, animals were placed into metabolic cages (Tecniplast Metabolic Cage System, Buguggiate, Italy) for 24 h to measure urine creatinine and protein concentrations by standard laboratory methods as described previously^35–37,52^ (Fig. 1). Blood was collected from the saphenous vein at week 4 and from the abdominal aorta at week 13 to measure serum carbamide (urea), creatinine, and lipid levels to verify the development of CKD^35–37,52^. Serum urea and creatinine levels were quantified by kinetic UV spectrophotometric method using urease and glutamate dehydrogenase enzymes and Jaffe’s method, respectively, using the Roche Diagnostics reagents and platform analyzers (Hoffmann-La Roche Ltd., Basel, Switzerland)^35,52^. At week 5 and the endpoint, urine creatinine and volume and serum creatinine concentration were measured^35,52^. Creatinine clearance, a renal functional indicator, was calculated using the standard formula (urine creatinine concentration [μM] × urine volume for 24 h [mL])/(serum creatinine concentration [μM] × 24 × 60 min)^40,52^. At weeks 5 and 13, total serum cholesterol and triglyceride levels were measured by Roche Cobas 8000 analyzer system using enzymatic colorimetric assays (Roche, Hoffmann-La Roche Ltd., Basel, Switzerland)^36^.

### Tissue harvesting

Thirteen weeks after the operations, rats were anesthetized with sodium pentobarbital administration (Euthasol; 40 mg/kg, *ip.*; Produlab Pharma b.v., Raamsdonksveer, The Netherlands) (Fig. 1)^36^. After blood pressure measurement, the abdominal cavity was opened to collect 1–1.5 mL blood from the aorta (see blood pressure measurement and serum and urine metabolite concentrations). After euthanasia by overdosed sodium pentobarbital (Euthasol, *ip.* 200 mg/kg; Produlab Pharma b.v., Raamsdonksveer, The Netherlands), the thoracic cavity was opened, the hearts were isolated, and the blood was washed out in a calcium-free Krebs–Henseleit solution. The total heart weight, and left and right ventricular weights (LV and RV, respectively) were measured. The LV was cut transversally as follows: the papillae ring was cut and fixed in 4% buffered formalin for histological analysis, while other parts were snap-frozen in liquid nitrogen and stored at − 80 °C until further analyses. Body weight, tibia length, left kidney weight, liver weight, and wet weight of lungs were measured at week 13^36^.

### Hematoxylin–eosin (HE) and picrosirius red and fast green (PSFG) stainings

Formalin-fixed paraffin-embedded subvalvular areas of the left ventricles were cut into 5 μm sections and stained with HE or PSFG as described previously^36,37^ (Fig. 1). Histological slides were scanned with a Pannoramic Midi II scanner (3D-Histech, Budapest, Hungary). Cardiomyocyte diameters, perimeters, and cross-sectional areas were measured on the digital HE images to verify the development of LVH at the cellular level^36,37,40^. Representative HE- and PSFG-stained slides were captured in Panoramic Viewer 1.15.4 (3D-Histech, Budapest, Hungary; https://old.3dhistech.com/pannoramic_viewer, last accessed on 14 May 2022)^36,37^.

Cross-sectional areas of 100 cardiomyocytes per LV sample were measured on HE images using the Biology Image Analysis Software (BIAS 1.0, Single-Cell Technologies Ltd., Szeged, Hungary, https://single-cell-technologies.com/bias/)^36,40,55,56^. Deep learning-based cytoplasm segmentation was performed after image pre-processing^36,40,55,56^. User-selected objects were forwarded to the feature extraction module configurable to extract properties from the selected cell components^36,40,55,56^.

Cardiac collagen content and fibrosis were assessed on PSFG slides with an in-house developed program as described previously^36,37^. Briefly, this program determined the proportion of red pixels in LV sections using two simple color filters. The first filter was used for detecting red portions of the image^36,37^. The second filter excludes any white (empty) or light grey (residual dirt on the slide) pixel from further processing using a simple RGB threshold^36,37^. Pixels in the first set corresponded to collagen and fibrosis^36,37^. Green pixels in the second set corresponded to cardiac muscle^36,37^. Dividing the number of elements in the first set by the number of elements in both sets gives the percentage of collagen in the cardiac area examined^36,37^.

### Cell culture experiments and RT-qPCR from human ventricular cardiac fibroblasts

Human ventricular cardiac fibroblasts (HVCFs, cryopreserved ampules of normal human ventricular cardiac fibroblasts containing ≥ 500,000 cells, #CC-2904, Lonza, Basel, Switzerland, https://bioscience.lonza.com/lonza_bs/CH/en/Primary-and-Stem-Cells/p/000000000000197234/NHCF-V-%E2%80%93-Human-Ventricular-Cardiac-Fibroblasts) were cultured in a fibroblast basal medium supplemented with 0.1% insulin, 0.1% fibroblast growth factor, 0.1% GA-1000, 1% pen-strep, and 10% FBS (all Lonza, Basel, Switzerland) as previously described^40,57^. Cultures were washed with HEPES buffered saline (Lonza, Basel, Switzerland) when indicated and split at a confluency level of 70%^40,57^. Cells were treated as follows: (i) without treatment—control; (ii) 20 ng/mL transforming growth factor-beta (TGF-β, R&D systems, Minneapolis, Minnesota, USA); (iii) 10 nmol/L P234; and (iv) 20 ng/mL TGF-β with 10 nmol/L P234 for 24 h. Then total RNA was extracted from HVCFs, and gene expressions of *Col1a1, Mmp9,* and *Acta2* were calculated relative to glyceraldehyde 3-phosphate dehydrogenase (*Gapdh*) and hypoxanthine–guanine phosphoribosyltransferase (*Hgprt*) expressions (the specific primer sequences and the description of the RT-qPCR method from HVCFs are detailed in the Supplementary Material).

### Transcription profiling by RT-qPCR from left ventricular samples

Total RNA was extracted from LV samples using the RNeasy Mini Kit (Qiagen, Hilden, Germany), quantified by NanoDrop spectrophotometer, and 100 μg of total RNA was reverse transcribed using the iScript cDNA Synthesis Kit (Bio-Rad Laboratories Inc., USA), as described previously^36,56^ (Fig. 1). Samples were analyzed in technical duplicates using a 10 μL reaction volume. The initial denaturation step of 3 min at 95 °C was followed by 40 cycles of 15 s 95 °C, 30 s 60 °C, and 40 s 72 °C, using a CFX-96 thermocycler with the accompanying CFX Manager software (Bio-Rad Laboratories Inc., USA) for relative quantification using the SQ values^36,55,56^. Specific primers (*Bax*: BCL2-associated X apoptosis regulator/apoptosis regulator BAX, #qRnoCED0002625; *Bcl2*: B-Cell CLL/lymphoma 2 apoptosis regulator, #qRnoCED0006419; *Casp7*: apoptosis-related cysteine peptidase, #qRnoCED00051028; *Col1a1*: collagen type 1 alpha 1 chain, #qRnoCED0007857; *Col3a1:* collagen type 3 alpha 1 chain, #qRnoCID0005033; *Ctgf*: connective tissue growth factor, #qRnoCED0001593; *Il1:* interleukin-1, #qRnoCID0002056; *Il6:* interleukin-6, #qRnoCID0053166; *Mmp9*: matrix metalloproteinase 9, #qRnoCED0001183; *Nppa*: A-type natriuretic peptide, #qRnoCED0006216; *Tgfb*: transforming growth factor-β, #qRnoCID0009191, *Tnf*: tumor necrosis factor-α, #qRnoCED0009117) and SsoAdvanced Universal SYBR Green Supermix (BioRad Laboratories Inc., USA) were used according to the manufacturer’s instructions^36,55,56^. Ribosomal protein lateral stalk subunit P2 (*Rplp2*) was the housekeeping control gene for normalization^55^. (The primer sequences of *Rplp2* are in the Supplementary Material).

### Western blot

The protein expression of BAX (20 kDa) and CASP7 (35 kDa) were quantified versus β-actin (45 kDa), while BCL-2 (26 kDa), ERK1/2 (42 and 44 kDa), pERK1/2 (42 and 44 kDa), KISS1R (40–140 kDa) were measured versus GAPDH (37 kDa) using a standard Western blot technique^55,56^ (Fig. 1). Briefly, LV samples (n = 28) were homogenized with an ultrasonicator (UP100H, Hielscher Ultrasonics GmbH, Germany) in RIPA buffer (#89900, Cell Signaling Technology Inc., Danvers, MA, USA) supplemented with phenyl methane sulfonyl fluoride (PMSF; Sigma-Aldrich, Saint Louis, MO, USA), sodium orthovanadate (Na_3_VO_4_; Sigma-Aldrich, Saint Louis, MO, USA) and sodium fluoride (NaF; Sigma-Aldrich, Saint Louis, MO, USA)^36,55,56^. The crude homogenates were centrifuged (15,000×*g*, 30 min, 4 °C)^36,55,56^. The supernatants’ protein concentration was quantified with the BCA Protein Assay Kit (Pierce Thermo Fisher Scientific Inc., Waltham, MA, USA)^36,55,56^, 50 μg of reduced and denaturized protein was loaded to analyze BAX and CASP7 proteins and 25 μg in the other cases. Then sodium dodecyl-sulfate (SDS) polyacrylamide gel electrophoresis (PAGE) was performed (50 V, 4 h, 10% gel), followed by protein transfer onto a nitrocellulose membrane (10% methanol, 35 V, 2 h in case of BCL2, ERK1/2, pERK1/2, and KISS1R, and 15% methanol, 35 V, 90 min in cases of BAX and CASP 7)^56^. The efficacy of transfer was checked using Ponceau staining^36,55,56^. The membranes were cut vertically and horizontally into parts corresponding to the molecular weight of each protein^36,55,56^. Membranes were blocked (1 h in 5% (w/v) bovine serum albumin [BSA, Saint Louis, MO, USA] supplemented with NaF and Na_3_VO_4_), and were incubated with primary antibodies: 1:500 against Anti-KISS1 Receptor (#AKR001, Alomone Labs, Jerusalem, Israel^58^), and BAX (#14796S, Cell Signaling Technology Inc., Danvers, MA, USA^59^); 1:1000 against CASP7 (#9492S, Cell Signaling Technology Inc., Danvers, MA, USA^60^), BCL2 (#196495, Abcam PLC, Cambridge, UK^61^), β-actin (#4970S, Cell Signaling Technology Inc., Danvers, MA, USA^60^), ERK1/2 (#4696S, Cell Signaling Technology Inc., Danvers, MA, USA^55^), pERK1/2 (#9911T, Cell Signaling Technology Inc., Danvers, MA, USA^55^) and 1:5000 against GAPDH (#2118, Cell Signaling Technology Inc., Danvers, MA, USA^36,55^) overnight at 4 °C in 5% BSA. The membranes were incubated with IRDye 800CW Goat Anti-Rabbit and/or IRDye 680RD Goat Anti-Mouse secondary antibody (LI-COR Biosciences, Lincoln, NE, USA, in the concentration of 1:20,000) for 1 h at room temperature in 5% BSA to reveal the primary antibodies^36,55,56^. Fluorescent signals were detected by Odyssey CLx machine (LI-COR Biosciences, Lincoln, NE, USA). Digital images were analyzed by densitometry using Quantity One Software (Bio-Rad Laboratories Inc., USA)^36,55,56^. The full-length Ponceau-stained membranes and corresponding Western blot images are shown in Supplementary Figs. S3–S8 (please find a more detailed description of our Western blot method in the supplementary materials in our previously published articles^36,56^).

### Statistical analysis

All analyses were performed with Sigmaplot 14.0 for Windows (Systat Software Inc., San Jose, CA, USA). All values are presented as mean ± S.E.M., p < 0.05 was accepted as a statistically significant difference. One-Way ANOVA was used to determine the statistical significance between all measured parameters within each time point. Two-way repeated-measures ANOVA was used to determine the effects of CKD and the treatments on serum, urine, and echocardiographic parameters between week 5 and endpoint follow-up data. Holm-Sidak test was used as a post hoc test. In cases of KISS1R, ERK1/2, and pERK1/2 Western blot results, an unpaired t-test was also used to investigate the statistical significance between CKD vs. sham-operated groups or P234-treated CKD vs. CKD-only groups. The corresponding Table or Figure legends describe specific sample numbers used for measurements.

### Supplementary Information

Supplementary Information.

## Data Availability

The datasets used and/or analyzed during the current study are available from the corresponding authors on reasonable request.
