# Supplementary material for: The kisspeptin-1 receptor antagonist peptide-234 aggravates uremic cardiomyopathy in a rat model
Source: Sci Rep. 2023 Aug 28;13:14046. doi: 10.1038/s41598-023-41037-0 (PMC10462750; doi:10.1038/s41598-023-41037-0)
Supplement: Supplementary file 1 — Supplementary Information. [file 41598_2023_41037_MOESM1_ESM.docx]

**Supplementary Material**

**The kisspeptin-1 receptor antagonist peptide-234 aggravates uremic cardiomyopathy in a rat model**

Hoa Dinh^1,2^ dinhhoaqa@gmail.com

Zsuzsanna Z.A. Kovács^1^ zsuzska.k93@gmail.com

Fanni Márványkövi^1^ marvanykovi.fanni@gmail.com

Merse Kis^1^ kissmerse@gmail.com

Klaudia Kupecz^1^ kupeczklau@gmail.com

Gergő Szűcs^1^ szucs.gergo@med.u-szeged.hu

Marah Freiwan^1^ marah.mf.94@gmail.com

Gülsüm Yilmaz Lauber^3^ guelsuem.yilmazlauber@meduniwien.ac.at

Eylem Acar^3^ eylem.acar@meduniwien.ac.at

Andrea Siska^4^ siska.andrea@med.u-szeged.hu

Katalin Eszter Ibos^5^ ibos.katalin.eszter@med.u-szeged.hu

Éva Bodnár^5^ eva.dobo4@gmail.com

András Kriston^6,7,8^ kriston.andras@single-cell-technologies.com

Ferenc Kovács^6,7,8^ kovacs.ferenc@single-cell-technologies.com

Péter Horváth^6,7,8^ peter.horvath@brc.hu

Imre Földesi^4^ foldesi.imre@med.u-szeged.hu

Gábor Cserni^9^ cserni.gabor@med.u-szeged.hu

Bruno K. Podesser^3^ bruno.podesser@meduniwien.ac.at

Peter Pokreisz^3^ peter.pokreisz@meduniwien.ac.at

Attila Kiss^3^ attila.kiss@meduniwien.ac.at

László Dux^1^* dux.laszlo@med.u-szeged.hu

Krisztina Csabafi^5#^ csabafi.krisztina@med.u-szeged.hu

Márta Sárközy^1,5^*^#^ sarkozy.marta@med.u-szeged.hu

^1^Department of Biochemistry and Interdisciplinary Centre of Excellence, Albert Szent-Györgyi Medical School, University of Szeged, H-6720, Szeged, Hungary

^2^Department of Biochemistry, Bach Mai Hospital, Hanoi, 100000, Vietnam

^3^Ludwig Boltzmann Institute for Cardiovascular Research at Center for Biomedical Research and Tranlationaly Surgery, Medical University of Vienna, Vienna, A1090, Austria

^4^Department of Laboratory Medicine, Albert Szent-Györgyi Medical School, University of Szeged, H-6720 Szeged, Hungary

^5^Department of Pathophysiology, Albert Szent-Györgyi Medical School, University of Szeged, Szeged, H-6720, Hungary

^6^Synthetic and Systems Biology Unit, Biological Research Centre, Eötvös Loránd Research Network, H-6726 Szeged, Hungary

^7^Single-Cell Technologies Ltd, Szeged, H-6726, Hungary,

^8^Institute for Molecular Medicine Finland (FIMM), University of Helsinki, 00014, Helsinki, Finland

^9^Department of Pathology, Albert Szent-Györgyi Medical School, University of Szeged, Szeged, H-6720, Hungary

*Correspondence: sarkozy.marta@med.u-szeged.hu; dux.laszlo@med.u-szeged.hu

^#^These authors contributed equally to the work

**Supplementary Method**

**RT-qPCR in human ventricular cardiac fibroblasts and primer sequences**

Total RNA was isolated from human ventricular fibroblasts (HVCFs, cryopreserved ampules of normal human ventricular cardiac fibroblasts containing ≥ 500,000 cells, #CC-2904, Lonza, Basel, Switzerland, https://bioscience.lonza.com/lonza_bs/CH/en/Primary-and-Stem-Cells/p/000000000000197234/NHCF-V-%E2%80%93-Human-Ventricular-Cardiac-Fibroblasts) using the RNeasy Plus Micro Kit (#74037, Qiagen, Hilden, Germany) and quantified by Tecan spectrophotometer Tecan Group Ltd (Tecan Group Ltd, Männedorf, Switzerland). cDNA was prepared using the QuantiTect reverse transcription kit (Qiagen, Hilden, Germany). Samples were analyzed in technical duplicates using a 20 μL reaction volume. The initial denaturation step of 3 min at 95 °C was followed by 40 cycles of 15 s 95 °C, 30 s 60 °C, and 30 s 72 °C, using a CFX Opus thermocycler with the accompanying software (Bio-Rad Laboratories Inc., USA) for Ct value analysis. Relative gene expressions of *Col1, Mmp9, and Acta2* calculated by the 2^−ΔΔCt^ method. Glyceraldehyde 3-phosphate dehydrogenase (*Gapdh*) and hypoxanthine-guanine phosphoribosyltransferase (*Hgprt)* were used as housekeeping control genes for normalization.

Primer sequences used in RT-qPCR experiments

| Gene symbol (species) | Forward primer sequence | Reverse primer sequence |
| --- | --- | --- |
| *Acta2* (human) | CCA GAG CCA TTG TCA CAC AC | CAG CCA AGC ACT GTC AGG |
| *Col1a1* (human) | AGT CGA GGG CCA AGA CGA AG | ACA ACA CCT TGC CGT TGT CG |
| *Mmp9* (human) | GAC GAC CGG TTT GGC TTC TG | GAG CTT GTC CCG GTC GTA GT |
| *Hgprt* (human) | TGA CAC TGG CAA AAC AAT GCA | AAG CTT GCG ACC TTG ACC AT |
| *Gapdh* (human) | TCC TGT TCG ACA GTC AGC CG | CCC CAT GGT GTC TGA GCG AT |
| *Rplp2* (rat) | AGC GCC AAA GAC ATC AAG AA | TCA GCT CAC TGA TGA CCT TGT T |

**Table S1** The Effects of the KISS1R antagonist peptide-234 on selected cardiovascular risk factors at week 13

| **Parameter (unit)** | **Sham** | **CKD** | **CKD + P234 D1** | **CKD + P234 D2** |
| --- | --- | --- | --- | --- |
| Serum cholesterol (mmol/L) | 1.57 ± 0.09 | 2.14 ± 0.07* | 2.61 ± 0.19* | 2.84 ± 0.33*# |
| Serum triglyceride (mmol/L) | 0.51 ± 0.09 | 0.76 ± 0.13 | 0.66 ± 0.07 | 0.93 ± 0.12*# |
| SBP (mmHg) | 140.2 ± 6.7 | 153.9 ± 6.6 | 171.1 ± 10.7* | 160.0 ± 10.4 |
| DBP (mmHg) | 98.6 ± 3.4 | 103.4 ± 4.6 | 113.4 ± 8.0 | 110.6 ± 7.2 |
| MBP (mmHg) | 113.2 ± 4.4 | 121.5 ± 5.1 | 133.2 ± 8.9 | 127.0 ± 8.7 |

Values are presented as mean ± S.E.M., * p < 0.05 vs. sham, # p < 0.05 vs. CKD (n = 7-8, one-way ANOVA, Holm-Sidak post hoc test). Sham: sham-operated group, CKD: chronic kidney disease group, CKD+P234 D1: chronic kidney disease group treated with the lower dose (13 μg/day, dose 1) of KISS1R antagonist peptide-234, CKD+P234 D2: chronic kidney disease group treated with the higher dose (26 μg/day, dose 2) of KISS1R antagonist peptide-234. DSP: diastolic arterial blood pressure, MBP: mean arterial blood pressure, SBP: systolic arterial blood pressure.

**Table S2** The Effects of the KISS1R antagonist peptide-234 on tibia length and organ weights in CKD at week 13.

| **Parameter (unit)** | **Sham** | **CKD** | **CKD+P234 D1** | **CKD+P234 D2** |
| --- | --- | --- | --- | --- |
| Body weight (g) | 449 ± 11 | 462 ± 10 | 444 ± 12 | 471 ± 13 |
| Heart weight (mg) | 1074 ± 24 | 1202 ± 17 * | 1244 ± 43 * | 1214 ± 56 * |
| LV weight (mg) | 779 ± 17 | 866 ± 16 * | 895 ± 30 * | 896 ± 37 * |
| RV weight (mg) | 206 ± 11 | 209 ± 7 | 215 ± 10 | 203 ± 14 |
| Lung weight (mg) | 1502 ± 21 | 1687 ± 28 * | 1732 ± 54 * | 1765 ± 31 *# |
| Liver weight (g) | 10.7 ± 0.3 | 11.2 ± 0.7 | 10.6 ± 0.4 | 13.4 ± 1.2* |
| Kidney weight (mg) | 1214 ± 21 | 1496 ± 83 * | 1437 ± 74 * | 1599 ± 101 * |
| Tibia length (cm) | 4.28 ± 0.09 | 4.26 ± 0.03 | 4.29 ± 0.04 | 4.36 ± 0.05 |
| LV/ Tibia length | 182 ± 4 | 203 ± 4 * | 209 ± 7 * | 206 ± 9 * |

Values are presented as mean ± S.E.M., * p < 0.05 vs. sham (n = 7-8, one-way ANOVA, Holm-Sidak *post hoc* test). Sham: sham-operated group, CKD: chronic kidney disease group, CKD+P234 D1: chronic kidney disease group treated with the lower dose (13 μg/day, dose 1) of KISS1R antagonist peptide-234, CKD+P234 D2: chronic kidney disease group treated with the higher dose (26 μg/day, dose 2) of KISS1R antagonist peptide-234. LV: left ventricle, RV: right ventricle.

**
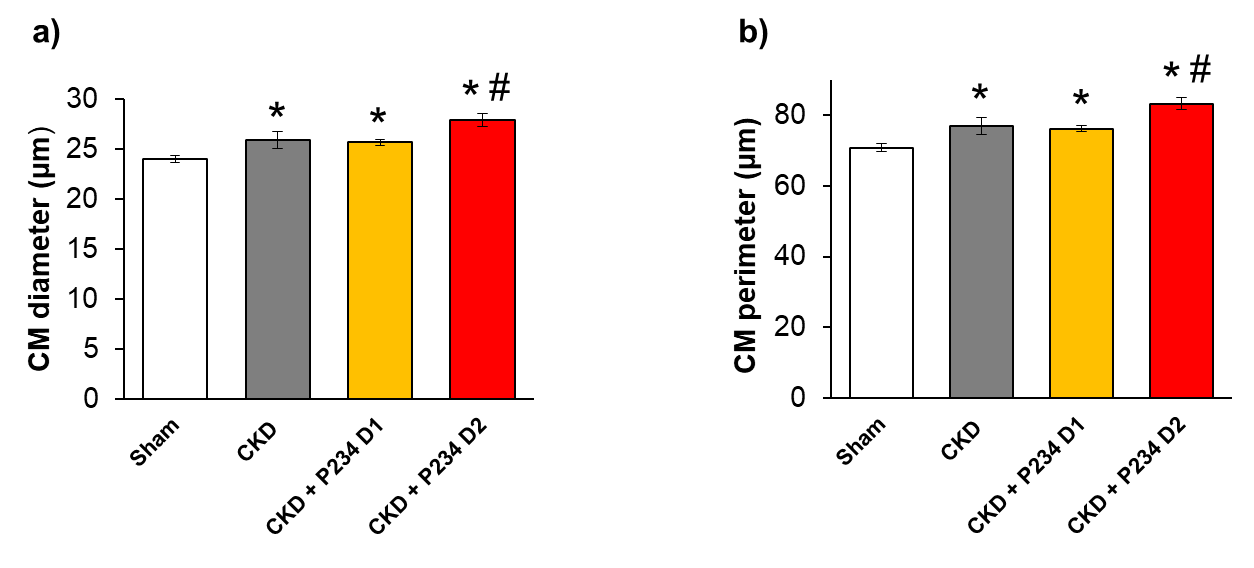
**

**Figure S1** The effects of the KISS1R antagonist peptide-234 on cardiomyocyte (CM) (a) diameter and (b) perimeter at week 13. On the digital HE images, cardiomyocyte diameters and perimeters were measured in 100 selected cardiomyocytes on left ventricular sections cut on the same plane. Values are presented as mean ± S.E.M., *p < 0.05 vs. sham, #p < 0.05 vs. CKD (n = 7–8, one-way ANOVA, Holm-Sidak post hoc test). Sham: sham-operated group, CKD: chronic kidney disease group, CKD + P234 D1: chronic kidney disease group treated with the lower dose (13 μg/day, dose 1) of KISS1R antagonist peptide-234, CKD + P234 D2: chronic kidney disease group treated with the higher dose (26 μg/day, dose 2) of KISS1R antagonist peptide-234.


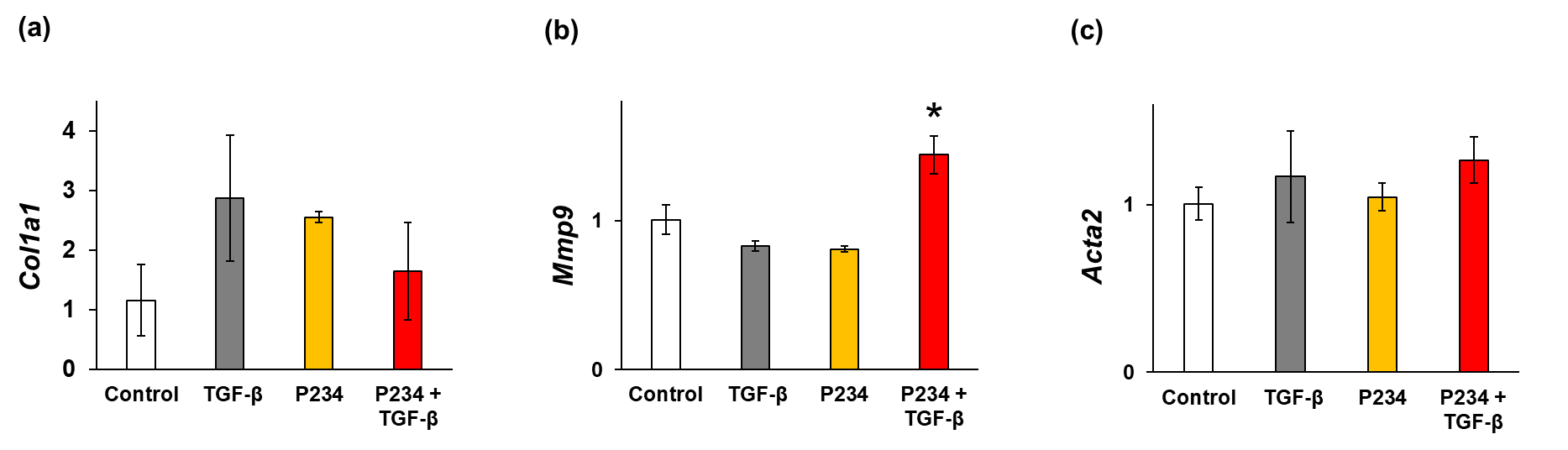


**Figure S2** The effects of P234 with or without TGF-β on the expression of collagen-1 (*Col1a1*)*,* matrix metalloprotease-9 (*Mmp9*)*,* and α-smooth muscle actin (*Acta2*) in human ventricular cardiac fibroblasts HVCFs. Values are presented as mean ± S.E.M., * p < 0.05 vs. sham (n = 3, One-Way ANOVA, Bonferroni *post hoc* test).


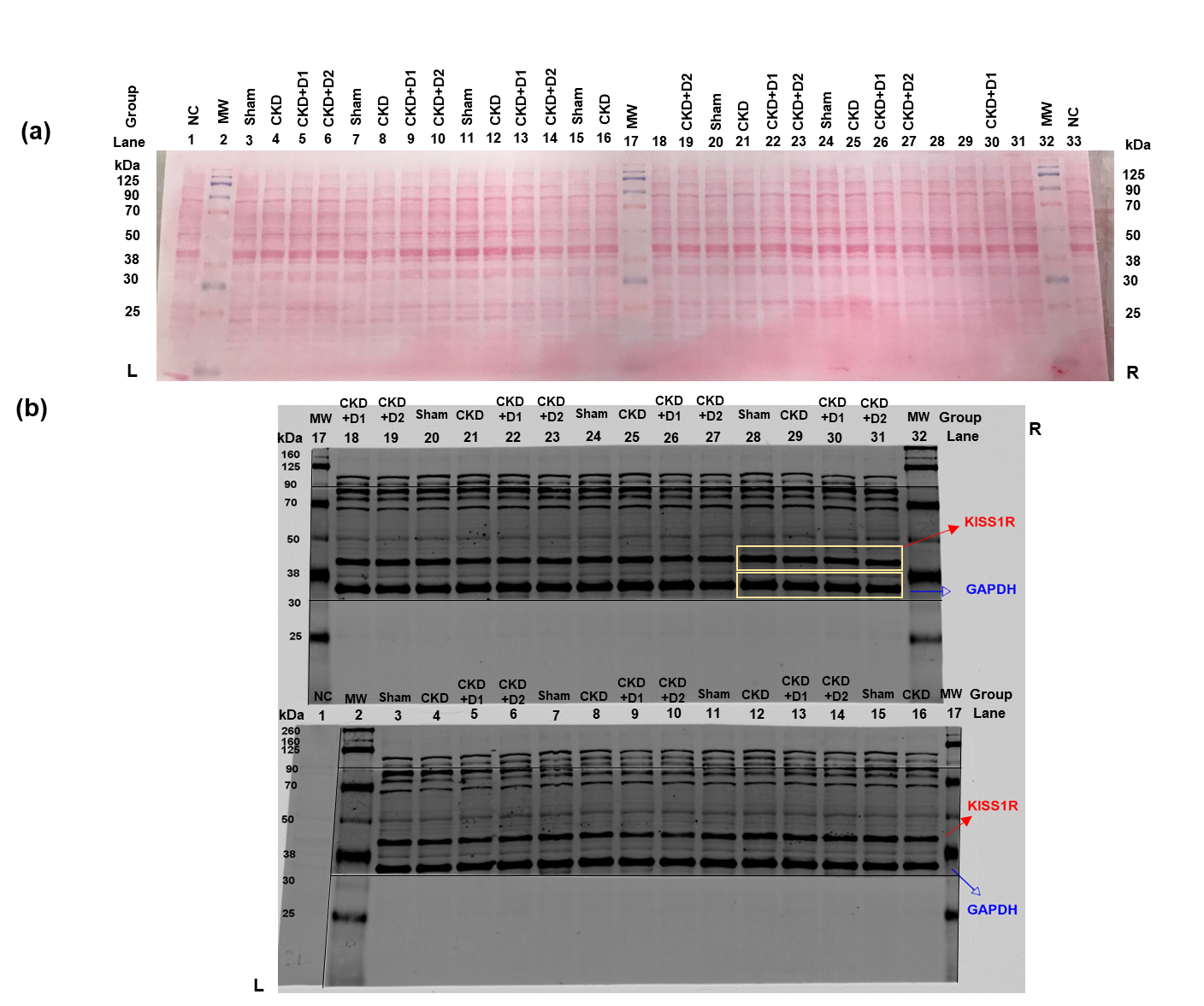


**Figure S3** Unmodified Ponceau-stained membranes and Western blot images of KISS1R and GAPDH. (a) Photo of the uncropped and unmodified Ponceau-stained membrane used later for the detection of KISS1R and GAPDH. The efficacy of the transfer of proteins onto a nitrocellulose membrane was checked using Ponceau staining. Images were captured by the camera of an Apple iPhone 7 plus. (b) Unmodified Western blot images of KISS1R and GAPDH. Images were captured with the Odyssey CLx machine and exported with Image Studio 5.2.5 software. Bands in yellow boxes are shown in Fig. 6 as representative blots. Sham: sham-operated group, CKD: chronic kidney disease group, CKD+D1: chronic kidney disease group treated with the lower dose (13 μg/day, dose 1) of KISS1R antagonist peptide-234, CKD+D2: chronic kidney disease group treated with the higher dose (26 μg/day, dose 2) of KISS1R antagonist peptide-234. MW: molecular weight marker, NC: negative control, L: left, R: right.

**
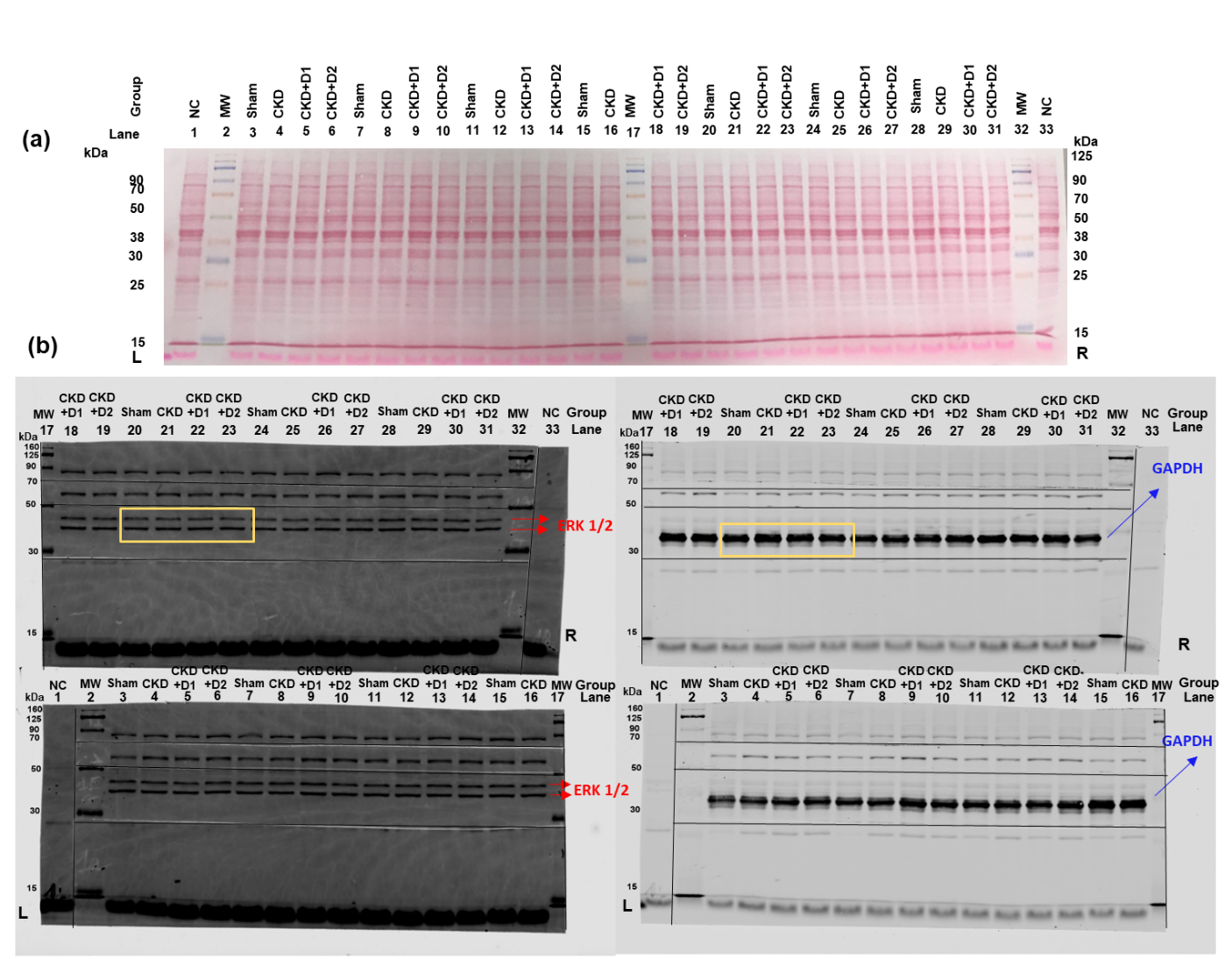
Figure S4** Unmodified Western blot images of ERK 1/2 and GAPDH. (a) Photo of the uncropped and unmodified Ponceau-stained membrane used later for the detection of ERK 1/2 and GAPDH. The efficacy of the transfer of proteins onto a nitrocellulose membrane was checked using Ponceau staining. Images were captured by the camera of an Apple iPhone 7 plus. (b) Unmodified Western blot images of ERK 1/2 and GAPDH. Images were captured with the Odyssey CLx machine and exported with Image Studio 5.2.5 software. Bands in yellow boxes are shown in Fig. 6 as representative blots. Sham: sham-operated group, CKD: chronic kidney disease group, CKD+D1: chronic kidney disease group treated with the lower dose (13 μg/day, dose 1) of KISS1R antagonist peptide-234, CKD+D2: chronic kidney disease group treated with the higher dose (26 μg/day, dose 2) of KISS1R antagonist peptide-234. MW: molecular weight marker, NC: negative control, L: left, R: right.

**
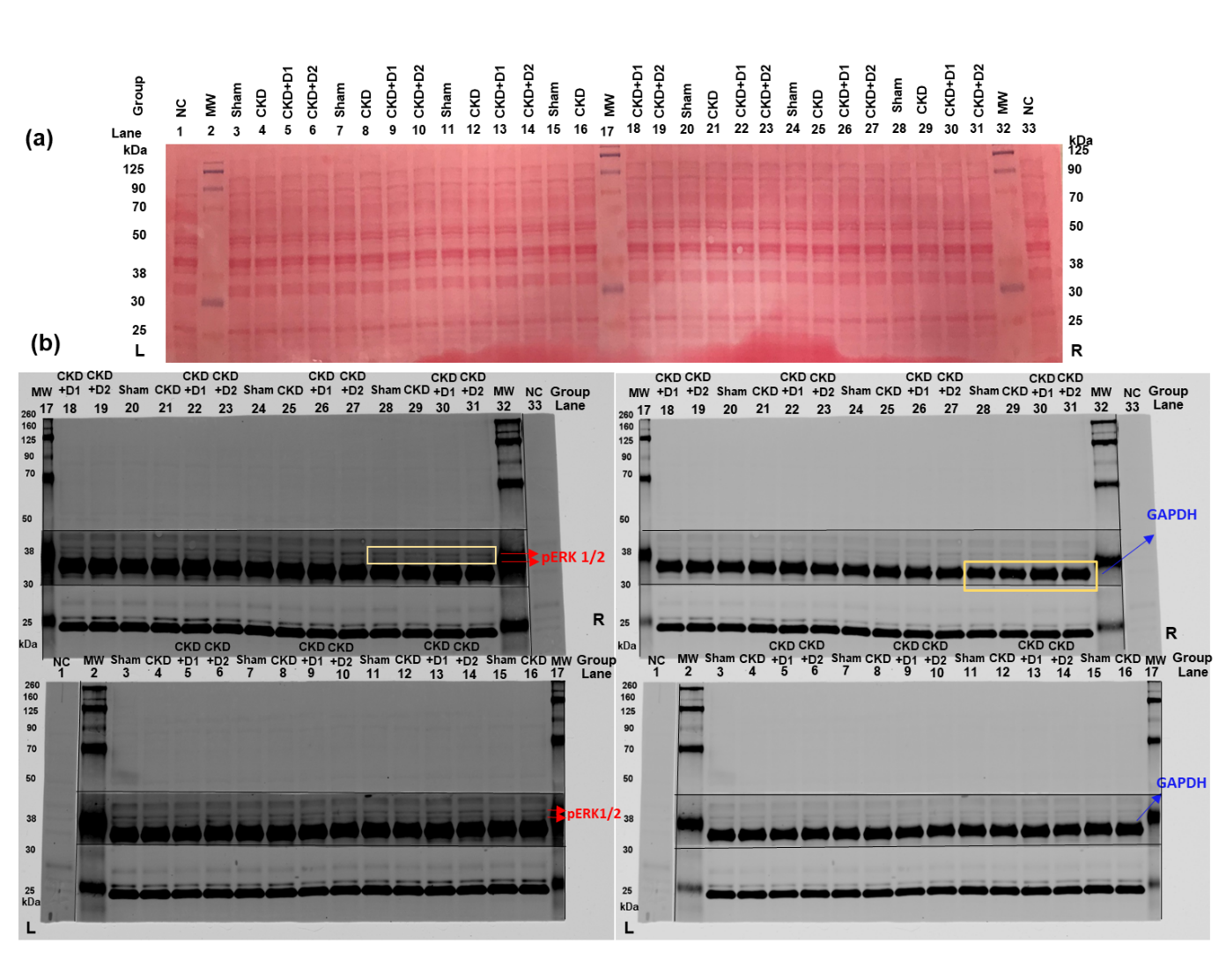
**

**Figure S5** Unmodified Western blot images of phospho-ERK 1/2 (pERK 1/2) and GAPDH**.** (a) Photo of the uncropped and unmodified Ponceau-stained membrane used later for the detection of pERK 1/2 and GAPDH. The efficacy of the transfer of proteins onto a nitrocellulose membrane was checked using Ponceau staining. Images were captured by the camera of an Apple iPhone 7 plus. (b) Unmodified Western blot images of pERK 1/2 and GAPDH**.** Images were captured with the Odyssey CLx machine and exported with Image Studio 5.2.5 software. Bands in yellow boxes are shown in Fig. 6 as representative blots. Sham: sham-operated group, CKD: chronic kidney disease group, CKD+D1: chronic kidney disease group treated with the lower dose (13 μg/day, dose 1) of KISS1R antagonist peptide-234, CKD+D2: chronic kidney disease group treated with the higher dose (26 μg/day, dose 2) of KISS1R antagonist peptide-234. MW: molecular weight marker, NC: negative control, L: left, R: right.


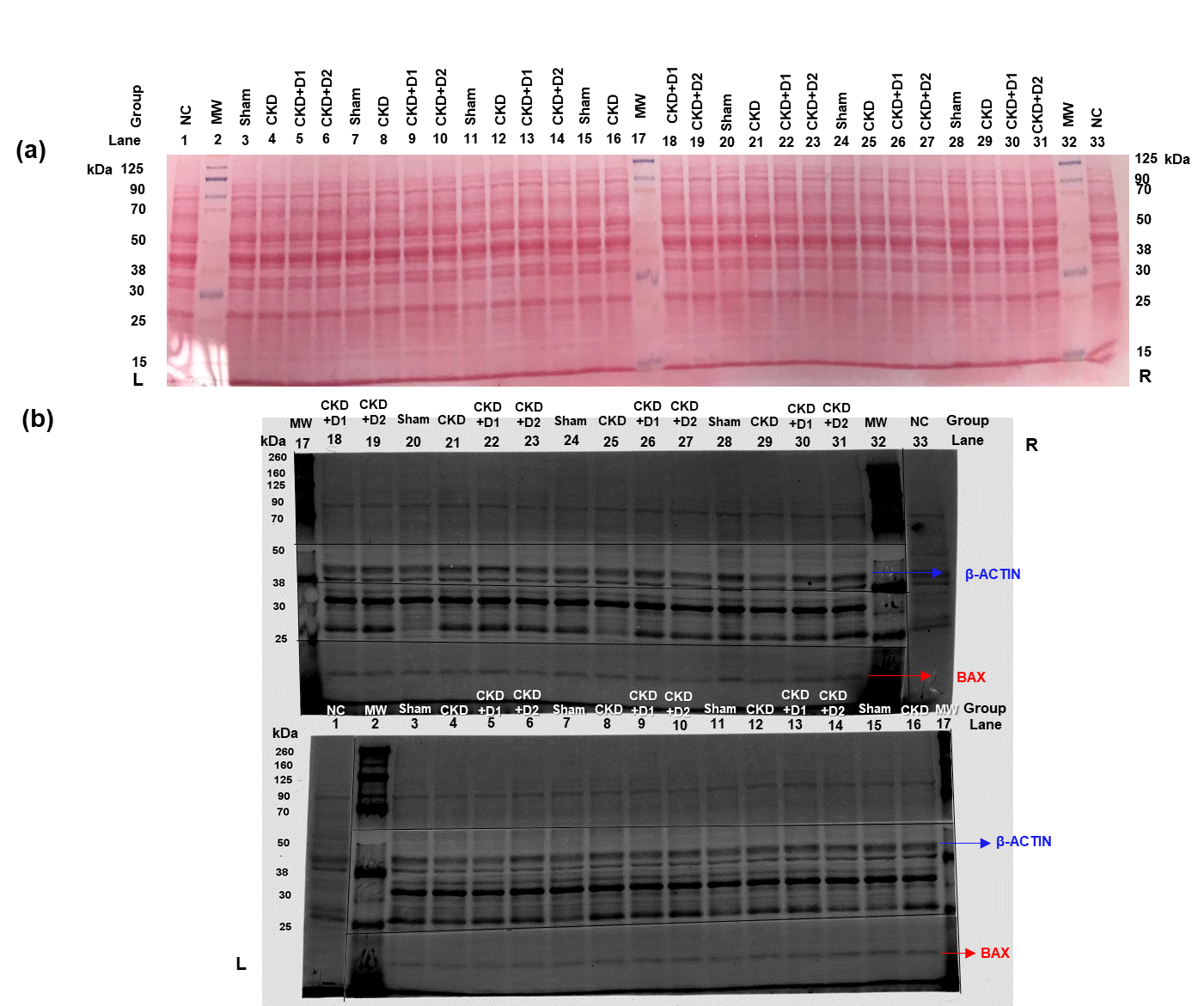


**Figure S6** Unmodified Western blot images of BAX and β-ACTIN. (a) Photo of the uncropped and unmodified Ponceau-stained membrane used later for the detection of BAX and β-ACTIN. The efficacy of the transfer of proteins onto a nitrocellulose membrane was checked using Ponceau staining. Images were captured by the camera of an Apple iPhone 7 plus. (b) Unmodified Western blot images of BAX and β-ACTIN. Images were captured with the Odyssey CLx machine and exported with Image Studio 5.2.5 software. Bands in yellow boxes are shown in Fig. 6 as representative blots. Sham: sham-operated group, CKD: chronic kidney disease group, CKD+D1: chronic kidney disease group treated with the lower dose (13 μg/day, dose 1) of KISS1R antagonist peptide-234, CKD+D2: chronic kidney disease group treated with the higher dose (26 μg/day, dose 2) of KISS1R antagonist peptide-234. MW: molecular weight marker, NC: negative control, L: left, R: right.

**
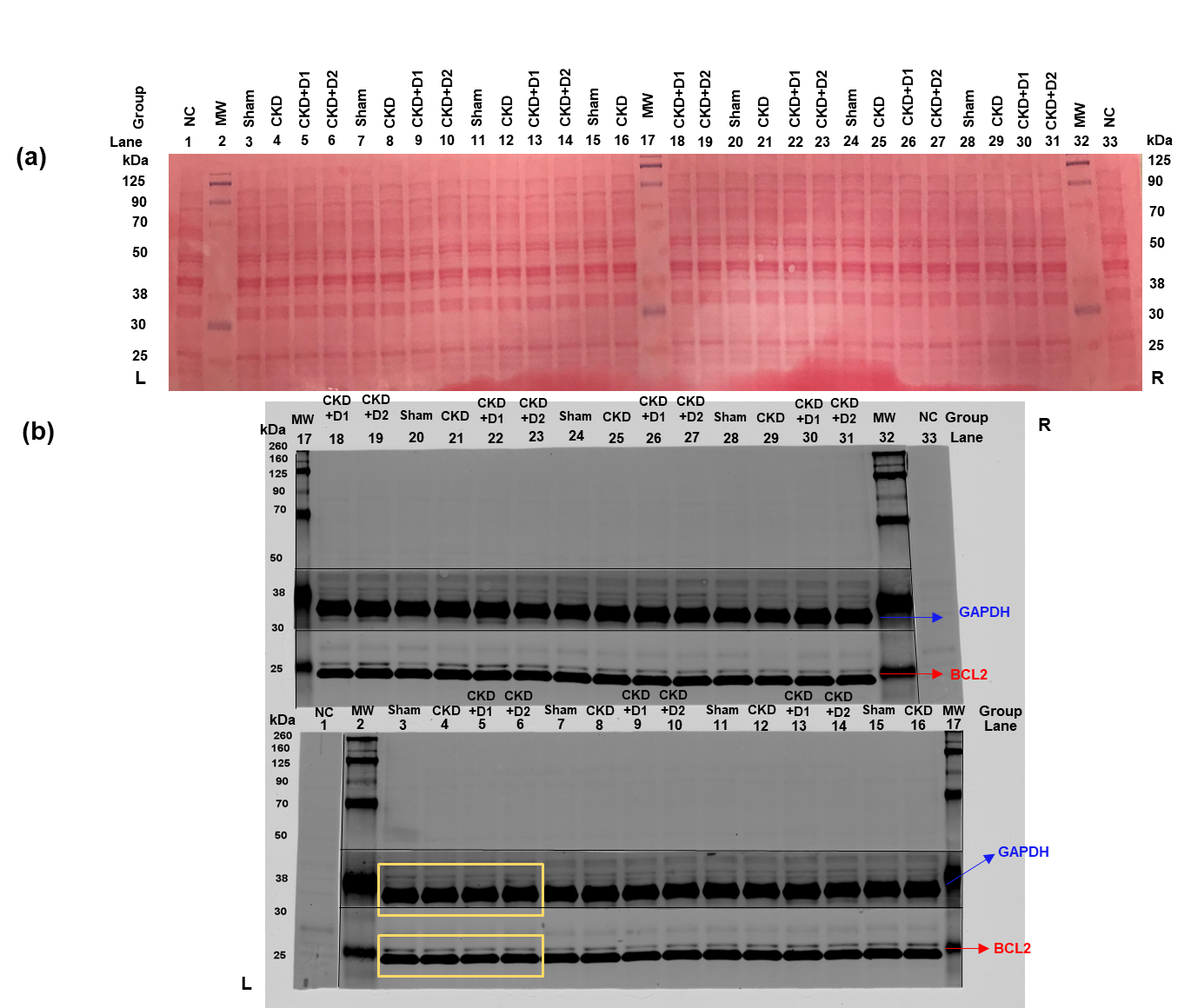
**

**Figure S7** Unmodified Western blot images of BCL2 and GAPDH. (a) Photo of the uncropped and unmodified Ponceau-stained membrane used later for the detection of BCL2 and GAPDH. The efficacy of the transfer of proteins onto a nitrocellulose membrane was checked using Ponceau staining. Images were captured by the camera of an Apple iPhone 7 plus. (b) Unmodified Western blot images of BCL2 and GAPDH. Images were captured with the Odyssey CLx machine and exported with Image Studio 5.2.5 software. Bands in yellow boxes are shown in Fig. 6 as representative blots. Sham: sham-operated group, CKD: chronic kidney disease group, CKD+D1: chronic kidney disease group treated with the lower dose (13 μg/day, dose 1) of KISS1R antagonist peptide-234, CKD+D2: chronic kidney disease group treated with the higher dose (26 μg/day, dose 2) of KISS1R antagonist peptide-234. MW: molecular weight marker, NC: negative control, L: left, R: right.

**
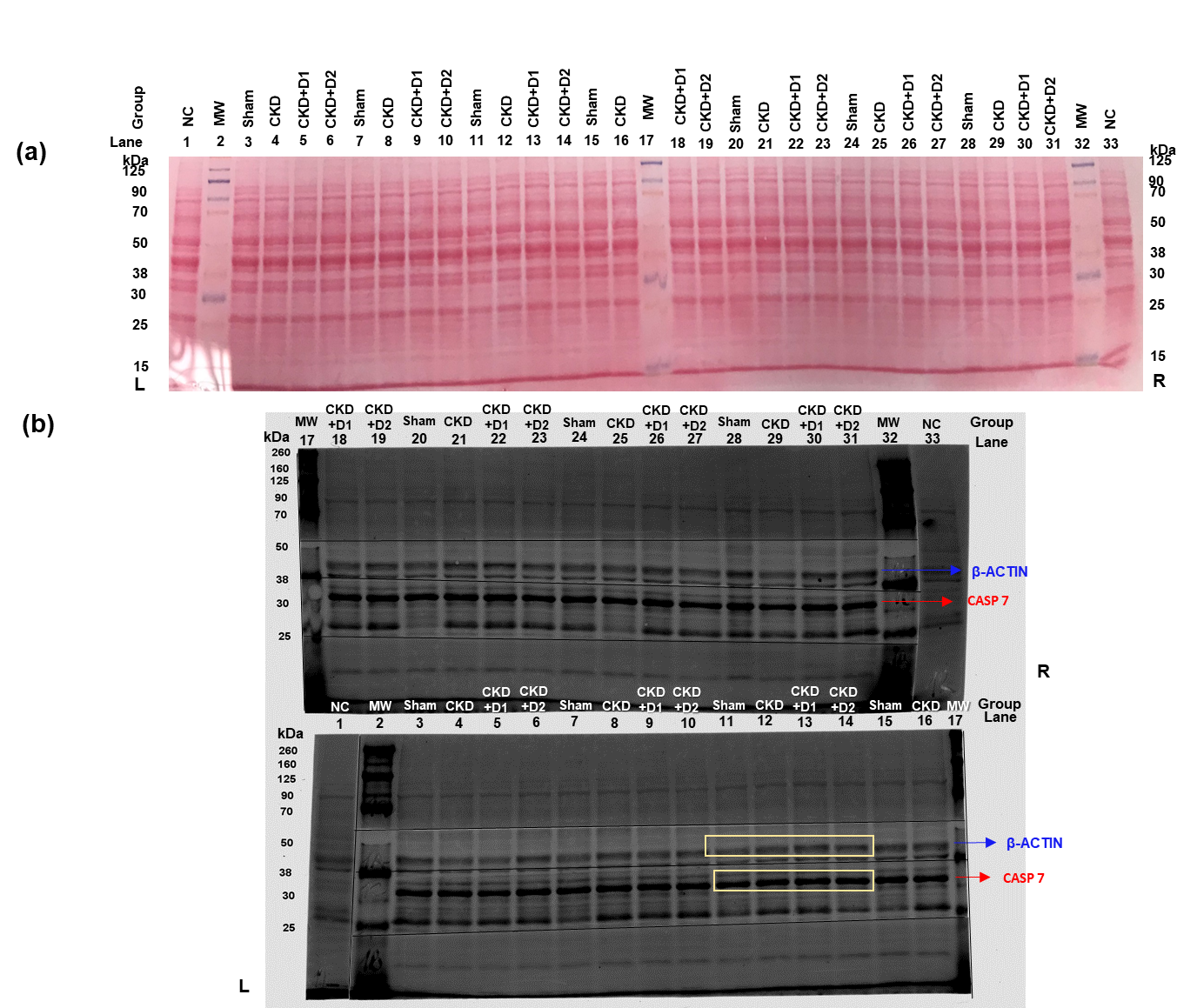
**

**Figure S8** Unmodified Western blot images of CASP 7 and β-ACTIN. (a) Photo of the uncropped and unmodified Ponceau-stained membrane used later for the detection of CASP 7 and β-ACTIN. The efficacy of the transfer of proteins onto a nitrocellulose membrane was checked using Ponceau staining. Images were captured by the camera of an Apple iPhone 7 plus. (b) Unmodified Western blot images of CASP 7 and β-ACTIN. Images were captured with the Odyssey CLx machine and exported with Image Studio 5.2.5 software. Bands in yellow boxes are shown in Fig. 6 as representative blots. Sham: sham-operated group, CKD: chronic kidney disease group, CKD+D1: chronic kidney disease group treated with the lower dose (13 μg/day, dose 1) of KISS1R antagonist peptide-234, CKD+D2: chronic kidney disease group treated with the higher dose (26 μg/day, dose 2) of KISS1R antagonist peptide-234. MW: molecular weight marker, NC: negative control, L: left, R: right.
